# The potential of liquid biopsy for detection of the KIAA1549-BRAF fusion in circulating tumor DNA from children with pilocytic astrocytoma

**DOI:** 10.1093/noajnl/vdae008

**Published:** 2024-01-24

**Authors:** Olha Krynina, Teresita Díaz de Ståhl, Cecilia Jylhä, Cecilia Arthur, Geraldine Giraud, Per Nyman, Anders Fritzberg, Johanna Sandgren, Emma Tham, Ulrika Sandvik

**Affiliations:** Department of Molecular Medicine and Surgery, Karolinska Institutet, Stockholm, Sweden; Department of Oncology-Pathology, Karolinska Institutet, Stockholm, Sweden; Department of Molecular Medicine and Surgery, Karolinska Institutet, Stockholm, Sweden; Clinical Genetics and Genomics, Karolinska University Hospital, Stockholm, Sweden; Department of Molecular Medicine and Surgery, Karolinska Institutet, Stockholm, Sweden; Clinical Genetics and Genomics, Karolinska University Hospital, Stockholm, Sweden; Department of Immunology, Genetic and Pathology, Neuro-oncology, and Neurodegeneration Program Rudbeck Laboratory, Uppsala, Sweden; Department of Women and Children’s Health, Akademiska University Hospital, Uppsala, Sweden; Department of Health, Crown Princess Victoria Children´s Hospital, Linköping University Hospital, Linköping, Sweden; Department of Medicine and Caring Sciences, Linköping University, Linköping, Sweden; Centre for Medical Image Science and Visualization (CMIV), Linköping University, Linköping, Sweden; Daycare Unit of Oncology and Hematology, Clinic of Pediatrics Falun Hospital, Dalarna Region, Sweden; Department of Oncology-Pathology, Karolinska Institutet, Stockholm, Sweden; Clinical Pathology and Cancer Diagnostics, Karolinska University Hospital, Stockholm, Sweden; Department of Molecular Medicine and Surgery, Karolinska Institutet, Stockholm, Sweden; Clinical Genetics and Genomics, Karolinska University Hospital, Stockholm, Sweden; Department of Clinical Neuroscience, Division of Neurosurgery, Karolinska Institutet, Stockholm, Sweden

**Keywords:** cerebrospinal fluid, cfDNA, ddPCR, *KIAA1549::BRAF*, pilocytic astrocytoma

## Abstract

**Background:**

Low-grade gliomas (LGGs) represent children’s most prevalent central nervous system tumor, necessitating molecular profiling to diagnose and determine the most suitable treatment. Developing highly sensitive screening techniques for liquid biopsy samples is particularly beneficial, as it enables the early detection and molecular characterization of tumors with minimally invasive samples.

**Methods:**

We examined CSF and plasma samples from patients with pilocytic astrocytoma (PA) using custom multiplexed droplet digital polymerase chain reaction (ddPCR) assays based on whole genome sequencing data. These assays included a screening test to analyze *BRAF* duplication and a targeted assay for the detection of patient-specific *KIAA1549::BRAF* fusion junction sequences or single nucleotide variants.

**Results:**

Our findings revealed that 5 out of 13 individual cerebrospinal fluid (CSF) samples tested positive for circulating tumor DNA (ctDNA). Among these cases, 3 exhibited the *KIAA1549::BRAF* fusion, which was detected through copy number variation (CNV) analysis (*n* = 1) or a fusion-specific probe (*n* = 2), while 1 case each displayed the *BRAF* V600E mutation and the *FGFR1* N577K mutation. Additionally, a quantitative analysis of cell-free DNA (cfDNA) concentrations in PA CSF samples showed that most cases had low cfDNA levels, below the limit of detection of our assay (<1.9 ng).

**Conclusions:**

While CNV analysis of CSF samples from LGGs still has some limitations, it has the potential to serve as a valuable complementary tool. Furthermore, it can also be multiplexed with other aberrations, for example, to the *BRAF* V600 test, to provide important insights into the molecular characteristics of LGGs.

**Key Points**

- We demonstrate that ddPCR can detect ctDNA from *KIAA1549::BRAF* fusion breakpoint sequences or other genomic aberrations in 5/13 CSF from PA patients.
- Copy number analysis of BRAF duplication with ddPCR assay can detect *KIAA1549::BRAF* fusion in rare cases and will gain more potential in combination with the *BRAF* V600 screening ddPCR test.

**Importance of the Study**

This study demonstrates potential applications and obstacles in implementing screening for the *KIAA1549::BRAF* fusion detection by ddPCR in cfDNA from the cerebrospinal fluid of pilocytic astrocytoma patients.

Central neural system tumors are the most common solid tumors in children, with considerable heterogeneity in localization, pathological characteristics, and clinical outcomes. The rapid development of the molecular characterization of cancer has expanded the known knowledge of the genetic aberrations that drive disease development and contributed to the most recent WHO tumor classification.^1^ Low-grade glial tumors (LGGs) prevail in the pediatric population and usually have single to few somatic molecular alterations. LGGs usually have excellent overall (OS) and progression-free survival (PFS), but when inoperable, both the treatment and the tumor growth cause significant morbidity, impacting these patients’ quality of life.

Many pediatric LGGs carry genetic alterations in the RAF/MEK/ERK (MAPK) pathway, specifically in *BRAF*. The most common aberration is the *KIAA1549::BRAF* fusion, which originates from a 2 Mb tandem duplication in 7q34. This fusion event juxtaposes the kinase domain of *BRAF* with the N-terminal region of *KIAA1549*, allowing the *BRAF* kinase domain to function independently from its N-terminal negative regulators.^2^ The chromosomal breakpoints are usually located in intronic regions and can form fusions with different combinations of exons where *KIAA1549* exon 15—*BRAF* exon 9 and *KIAA1549* exon 16—*BRAF* exon 9 are the most common.^3^ Patients with *BRAF* fusion are, like all low-grade gliomas, primarily operated on and receive standard chemotherapy if the tumor is nonoperable or progressive after resection. They have a very good 10-year PFS of 67% and OS of 98%,^4,5^ which makes detecting this aberration beneficial for reliable risk stratification and prognosis. Furthermore, the BRAF fusion also confers a possibility for targeted therapy using BRAF inhibitors, sometimes combined with downstream MEK inhibitors with a better effect than standard treatment.^6,7^ The second most reported genomic aberration in pediatric LGGs is the *BRAF* V600E amino acid substitution. Tumors with this mutation have a more malignant phenotype and exhibit less favorable survival rates, with a 10-year PFS of around 35% and OS of 90%.^4,5^ It is necessary to distinguish these patients by molecular characterization since they can be treated with specific RAF inhibitors such as vemurafenib or dabrafenib and achieve a better response.^8^ Also, current protocols may include a combination of RAF and MEK inhibitors to overcome resistance development to RAF inhibitors and improve clinical outcomes.^9^

The *KIAA1549::BRAF* fusion mainly occurs in pilocytic astrocytoma (70–80%), but it has also been reported in pilomyxoid astrocytoma (PMA) and ganglioglioma patients.^10^ and up to 60% of diffuse leptomeningeal glioneuronal tumor (DLGNT) patients with subtentorial and spinal tumor localization also exhibit *KIAA1549::BRAF* fusion.^11^ Molecular diagnosis is established through analysis of CNS tissue biopsies. Classical methods of *KIAA1549::BRAF* fusion detection include analysis by fluorescence in situ hybridization, RNA sequencing, or RT-PCR.^12^ Recent studies have applied several high-throughput genomic techniques such as NGS and proved their efficiency in retrieving genomic information from PA tumor tissue samples.^12–14^ For example, custom gene panel sequencing data can be used to generate copy number profiles that can be assessed for focal copy number gain on 7q34.^13^ Whole genome sequencing can provide the most comprehensive information about a patient’s genome, including the genomic location of fusion breakpoint, but it is not available for everyone worldwide.^15^ These methods were applicable to tumor genomic DNA and needed decent amounts of input DNA.

Over the past years, liquid biopsy has shown its potential as a convenient source of circulating tumor DNA (ctDNA). Molecular characterization of ctDNA is slowly entering clinical practice and can be applied for diagnostic and longitudinal minimal residual disease (MRD) monitoring for different types of cancers.^16^ Cerebrospinal fluid (CSF) ctDNA has become the center of attention for brain tumors. Recently, the utility of sequencing (NGS) and ddPCR methods to detect ctDNA in CSF samples has been demonstrated, that is, in high-grade gliomas^17^ and medulloblastoma.^18^ For LGGs, ctDNA analysis can be challenging and requires highly sensitive techniques since the likelihood of detection of ctDNA correlates with active disease and high tumor burden.

Targeted determination of hotspot mutations and genomic aberrations in tumor gDNA or cfDNA can be achieved with droplet digital PCR (ddPCR). ddPCR requires minimal DNA input and provides absolute quantification of as little as 3–5 haploid genome equivalents (hGEs) in diagnostic or MRD liquid biopsy samples.^18^ Successful application of ddPCR for *KIAA1549::BRAF* detection was demonstrated by Appay et al.^19^ They implemented a copy number variation (CNV) analysis approach and showed its feasibility using a low concentration of input genomic DNA with a low tumor DNA fraction.

As CNS tissue biopsies are not always available or risk-free to perform (and can lead to long-term complications), we sought to explore the potential of ddPCR to quantify ctDNA in liquid biopsies from LGGs. We aimed to test the possibility of screening for the *KIAA1549::BRAF* fusion by assessing copy number changes of a duplicated region of *BRAF*. Simultaneously, we complemented this approach with patient-specific assays for detecting fusion gene junction sequences or SNVs.

## Materials and Methods

### Patients’ Characteristics

Among a larger cohort of consecutive pediatric brain tumor patients collected since 2018 at the Karolinska University Hospital and since 2023 also from Akademiska University Hospital in Uppsala and Linköping University Hospital, we identified 16 patients with pilocytic astrocytoma. The study was approved by the Swedish Ethical Review Authority (2016/2-31/1, 2016-03-09; 2017/599-32/1, 2017-03-28; 2018/1472-32/1, 2018-07-13; 2019-01222, 2019-04-12; 2016/429-31/2, 2016-04-18; 2018/1484-32/2, 2018-08-15; 2019-01221, 2019-04-15). The participants’ legal guardians gave their written informed consent. As normal controls (NC), we included 2 patients with brain malformation and epidermoid cyst that required ventriculoperitoneal shunting due to hydrocephalus.

### Sample Collection and Processing

All CSF samples were collected if CSF was available as part of the clinical routine. CSF and plasma samples were collected in Cell-Free DNA (cfDNA) BCT tubes (STRECK, La Vista, NE, USA). Liquid biopsies, stored at room temperature for no more than 9 days, were double centrifuged at 4°C at 1600*g* and 16,000*g* to remove cellular components. Supernatants were frozen at –80°C for future use. CfDNA was extracted with QIAamp Circulating Nucleic Acid Kit on the QIAvac24 Plus vacuum manifold using the supplier’s protocol. cfDNA was eluted in 40 µl of AVE buffer from the kit. DNA concentration was measured using a Qubit 4 Fluorometer (Thermo Fisher Scientific) with the High Sensitivity Double-Stranded DNA kit. Extracted cfDNA was kept frozen at −20°C for further analysis. CfDNA from plasma from blood donors was extracted in the same way for use as normal plasma cfDNA controls. Normal control (NC) genomic DNA (gDNA) was extracted from the peripheral blood of noncancer patients with QIAamp DNA Blood Maxi kit (Qiagen, Hilden, Germany) and anonymized and pooled in 1 sample.

### Whole Genome Sequencing of Tumor gDNA

Tumor gDNA samples were available from The Swedish Childhood Tumor Biobank^20^ for all Karolinska University Hospital patients. Isolation of gDNA from tumors was performed using the AllPrep DNA/RNA/Protein Mini kit (Qiagen, Hilden, Germany) and from blood (minimum 1 ml) using the QIAamp® DNA Blood Midi/Maxi kit, vacuum protocol (Qiagen), according to the manufacturer’s instructions. WGS analysis was performed on paired tumor-normal data by The Swedish Childhood Tumor Biobank as previously described and used to detect driver genetic aberrations.^18^

### Digital Droplet PCR Assay Development

The development of a BRAF duplication screening ddPCR assay was based on a previously published approach,^19^ but in our work, we focused on *BRAF* exons 3 and exon 15. Exon 15 includes valine at the 600 positions, the location of the *BRAF* hotspot mutation. To create custom targeted ddPCR assays for subsequent multiplexing with a screening test, we retrieved from WGS data 300 bp DNA sequences around positions of predetermined driver aberrations. Primers and probes were purchased from BioRad (Hercules, CA, USA) or designed manually using the IDT (Integrated DNA Technologies, Coralville, IA, USA) Primer Quest tool (Supplementary Table 1). Primer-BLAST tool (NCBI) was used to check for specificity and analysis of possible dimers and secondary structure formations. Amplicon sizes were set to be in the range of 75–120 bp. Target probes for mutant sequence detection were labeled with a 5´ 6-FAM™ fluorophore and a 3´ Iowa Black^®^FQ quencher. *BRAF* exon 3 and exon 15 probes were labeled with a 5´ HEX fluorophore and a 3´—Iowa Black^®^FQ quencher. For *BRAF* V600E/K/R mutation detection, we used a commercially available BRAF screening assay from BioRad.

First, we performed temperature gradients for all assays in single plex using normal and positive control gDNA as a template. For a screening *BRAF* duplication assay, different concentrations of primer/probe mix for exons 3 and 15 were tested for amplitude multiplexing to find the best resolution between different clusters of droplets. Screening *BRAF* duplication and custom fusion breakpoint assays were combined in multiplexed ddPCR assays. To determine the limits of detection (LoD) of *BRAF* CNV detection using a multiplexed ddPCR assay, we performed a 2-fold series of dilutions of 1 sample of tumor gDNA starting from 5 ng. The LoD represents the minimal gDNA concentration with a significant difference between CNV value for normal and tumor samples without overlapping error bars.

All ddPCR reactions were run on the QX200 AutoDG Droplet Digital PCR System/QX200 Droplet Reader with 4X ddPCR Multiplex Supermix for probes (no dUTP) (BioRad) according to the manufacturer’s instructions. Results were analyzed manually using the BioRad QX Manager Software 2.0 Standard Edition. Sample were considered positive for fusion or SNV ctDNA if there were at least 3 detected copies of the mutated sequence.

### Patients’ cfDNA Analysis With ddPCR Assays

cfDNA extracted from CSF and plasma samples were analyzed using either multiplexed ddPCR assays or specific targeted ddPCR assays. cfDNA samples were run in triplicates with 11 µl of eluted cfDNA per well. Plates were run with additional controls in 3 wells each that included patient-matched gDNA positive control (4 ng tumor DNA/well), NC gDNA (4 ng/well), NC CSF cfDNA, and NC plasma cfDNA (11 µl/well) in 9 wells to ensure accurate detection of ctDNA. ddPCR results were analyzed manually with BioRad QX Manager, where plots with concentrations of copies, copy numbers, ratio, and fractional abundance for mutated sequences were generated. The final cfDNA amount (yield) extracted from patients’ samples was calculated by multiplication of the total number of cfDNA copies by 3.3 pg (mass of haploid genome) and corrected for the fraction of the elution volume used in the ddPCR experiments (33 of 40 µl). Summarized scatter plots and statistical analysis were performed with GraphPad Prism 8.0.

## Results

### Characterization of Cohort of PA Patients

For this study, we included 16 pediatric patients aged 5 months to 14 years with a primary diagnosis of pilocytic astrocytoma (Table 1). Fourteen of these patients were selected after the primary diagnosis was established at Karolinska University Hospital by MRI examination, during the years 2018–2022. Patients 58 and 62 were diagnosed and treated at Falu Lasarett in collaboration with Akademiska Hospital and Linköping University Hospital, respectively. Patients 17 and 58 relapsed 2 years after the primary disease and were included in the project at this stage. The diagnosis of patient 58 at relapse was changed from pilocytic astrocytoma to DLGNT since the MRI examination at relapse revealed leptomeningeal spreading in the mesencephalon, cerebellum, and hippocampus. Three patients had inoperable tumors due to diffuse infiltrative growth or location but were eligible for tissue biopsy. From 1 patient (patient 58), no tumor tissue was available. All patients have continued follow-up examinations until now.

**Table 1.** Clinical data and information on WGS results and CSF samples when available. The larger CNVs were detected on WGS data from tumor DNA.

UPN	Age	Tumor size (cm^3^)	Anatomical location	In contact with CSF	Method of CSF collection	Genetic aberrations in tumor	Larger CNVs	Relapse (Y/N)
11	9 years	3.5 × 2.5 × 4	4th ventricle	y	–	*KIAA1549::BRAF* fusion	chr 5,6 gain	Y
30	12 years	5.5 × 5 × 5	Vermis and cerebellum	y	Surgery	*KIAA1549::BRAF* fusion	–	N
32	3 years	5.5 × 4 × 4.5	Right cerebellar hemisphere	n	Surgery	*KIAA1549::BRAF* fusion	–	N
33	6 years	5.5 × 5 × 4	Vermis, 4th ventricle	y	Surgery	*KIAA1549::BRAF* fusion	–	N
10	8 years	6 × 1.5 × 2	Medulla oblongata, C4	y	Biopsy	*KIAA1549::BRAF* fusion	–	N
45	14 years	4 × 4.5 × 5	4th ventricle	y	Surgery	*KIAA1549::BRAF* fusion	–	N
46	7 years	3 × 3 × 2	4th ventricle	y	Surgery	*KIAA1549::BRAF* fusion	chr 7 gain	N
12	7 years	7 × 7 × 6	Supratentorial	n	–	*KIAA1549::BRAF* fusion	–	N
36	10 years	6.5 × 6.5 × 3	Frontal skull base	y	Biopsy	*FGFR1* N577K, *PTPN11* E76K)	chr 12 gain	N
3	13 years	4.6 × 4.7 × 5.4	Cerebellum	y	Surgery	*KIAA1549::BRAF* fusion	chr 5,6,7,11,12 gain	N
7	12 years	3 × 3 × 2.5	Right globus pallidus	n	Surgery	*KIF21B::NTRK1*, *NOS1AP::KIF21B* fusions	–	N
8	10 years	7 × 5 × 4	4th ventricle	y	Surgery	*KIAA1549::BRAF* fusion	–	Y
17	5 years	4 × 4.5 × 5	Cerebellum	y	Surgery	*KIAA1549::BRAF* fusion	–	Y
25	2 years	1 × 1 × 1	Cerebellum	n	–	*KIAA1549::BRAF* fusion	–	N
58	11 months	3,5 × 2,5 × 4	Spinal, leptomeningeal spreading mesencephalon, cerebellum, hippocampus	y	Lumbar puncture	N/A	N/A	Y
62	5 months	4.5 × 3.5 × 3	Supratentorial/suprasellar	n	VP-Shunt	*BRAF* V600E	N/A	N

CSF samples were collected during biopsy or surgery before tumor resection to prevent contamination of cfDNA with tumor gDNA. For patient 58, CSF was collected only at relapse via lumbar puncture and with no surgery or biopsy performed at this time point. From patient 62, CSF was collected during ventriculoperitoneal shunting. Plasma samples were collected before the operation or several days after during clinical examinations. CSF samples for normal cfDNA controls were collected from patients with nononcological hydrocephalus.

Comprehensive molecular characterization of tumors was performed for 14/16 patients using WGS on patients with matched tumors and normal samples. Sequencing results revealed the characteristic pattern for low-grade tumors, with a single to a few mutation somatic events and whole chromosomal CNVs, all listed in Table 1 (Supplementary Figure 1). 12/16 patients harbored a *KIAA1549::BRAF* fusion, and all of them had a 1-16/9-18 exon combination with unique intronic breakpoints on the genomic level. Patient 7 had a translocation of a 5 Mb fragment that includes exon 10 of *NTRK1* and exon 2 of *NOS1AP* within chromosome 1, forming an in-frame *KIF21B::NTRK1* fusion with a reciprocal *NOS1AP::KIF21B* fusion. Two pathogenic SNVs were detected in patient 36: *FGFR1* N577K and *PTPN11* E76K. Both these noncanonical pilocytic astrocytoma alterations have been previously described.^21,22^ A *BRAF* V600E mutation was detected by routine histopathology analysis in patient 62. No other SNVs of known clinical relevance were detected in our cohort of PA patients.

### Design and Optimization of Multiplexed ddPCR Assays

In the first step, we designed a screening assay using reference *BRAF* exon 3 and *BRAF* exon 15 to detect *BRAF* duplication status through CNV analysis. In addition, targeted ddPCR assays for each patient with a confirmed fusion were created based on the junction sequences from WGS data. The optimized *BRAF* duplication screening assay was successfully multiplexed with different patient-specific targeted assays, enabling simultaneous assessment of tumor DNA fraction and *BRAF* CNV in patient samples. Most multiplexed ddPCR assays had a false positive rate (FPR) of 0 (Supplementary Table 1). For patient 36, the *FGFR1* assay worked well, but the *PTPN11* assay demonstrated a high nonspecific background at working temperatures for the *FGFR1* assay and thus was not used for multiplexing and subsequent analysis.

**Figure 1.**
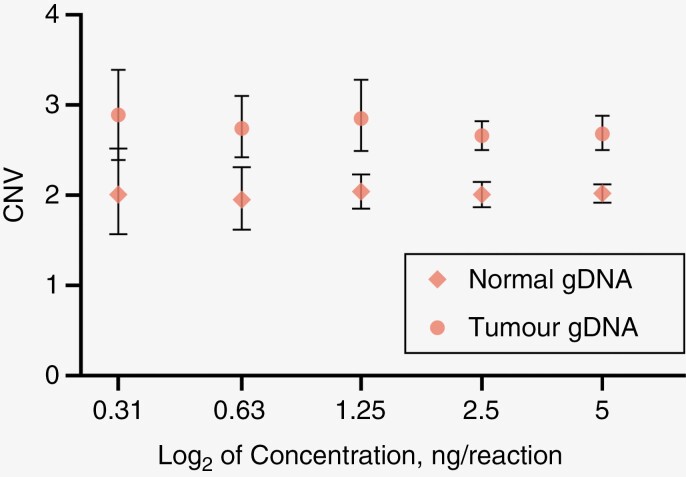
Evaluation of limits of detection of *BRAF* exon 15 duplication in multiplexed ddPCR assay. We performed a series of dilutions of gDNA extracted from blood cells (normal gDNA) and a tumor tissue biopsy sample from patient 11 (tumor gDNA) starting from 5 ng input per well (all wells in triplicate). The plot represents calculated CNV values for each input gDNA concentration. The lowest concentration where we did not see overlapping error bars is 0.63 ng/reaction (1.9 ng of DNA for analysis).

We performed a series of tumor gDNA dilutions using gDNA from patient 11 with 33% *BRAF* fusion as ascertained by ddPCR using a fusion-specific probe. The results confirmed that a minimum of 1.9 ng of DNA (0.63 ng of DNA per reaction, in triplicate) is needed for reliable confirmation of *BRAF* duplication based on significant differences between CN values calculated for normal and positive gDNA (Figure 1, Supplementary Figure 2), similar to the detected limit in a previously published assay.^19^

### Analysis of cfDNA With Multiplexed ddPCR Assays

**Figure 2.**
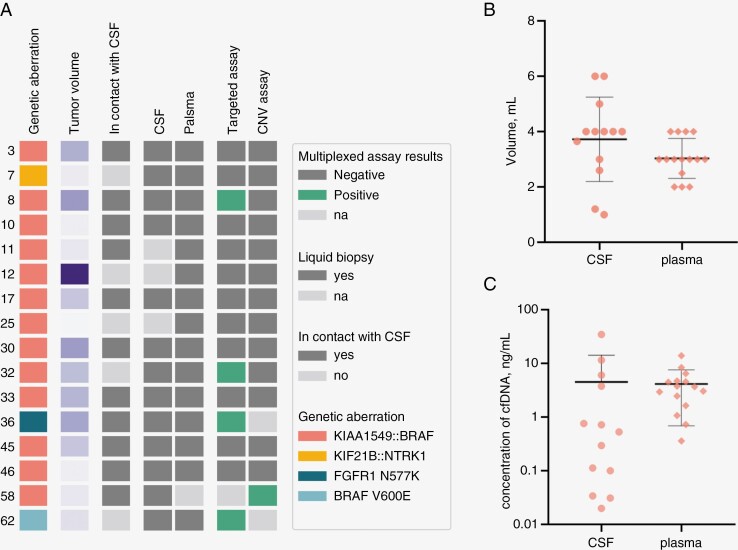
Liquid biopsy samples and ddPCR assays for the research cohort. (A) The color table summarizes the findings from all patients in the research cohort. Unique patient numbers are indicated on the left with the corresponding genetic aberrations. The approximate tumor volume varies in range from 1 to 295 cm^3^, where a darker color represents a bigger volume. The type of sample and the assays performed are also indicated. (B) Dot plot of volumes of liquid biopsy samples used for cfDNA extraction. (C) Dot plot of the concentrations of cfDNA in 1 ml of source material. The values were calculated based on the amount of *BRAF* exon 3 copies detected after ddPCR analysis.

For our study, CSF samples were available for 13/16 patients, and plasma was collected from 15/16 patients (Figure 2A). Total volumes of CSF used for cfDNA extraction varied from 1 to 6 ml; the range for plasma samples was between 2 and 4 ml (Figure 2B). We ran multiplexed ddPCR assays combining screening of the *BRAF* duplication and targeted fusion breakpoint assays for 13 patients with detected fusions. The CSF sample from patient 58 was tested only with the *BRAF* duplication screening assay. Liquid biopsy samples from patients 36 and 62 were examined with specific targeted SNV assays (Figure 2A).

**Figure 3.**
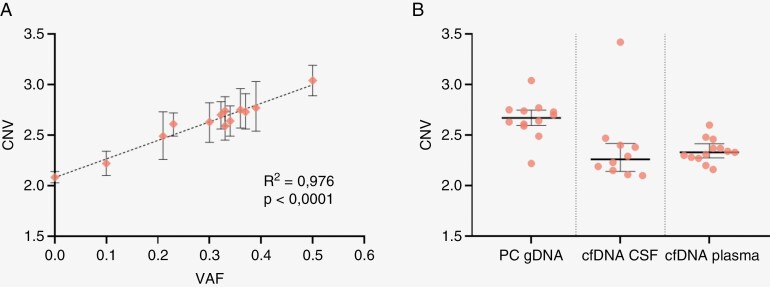
(A) Correlation of variant allele frequencies (VAFs) obtained using fusion junction sequence probe (x-axis) and CNV value for BRAF exon15/exon3 (y-axis) for ddPCR assay validation. 13 assays were tested with tumor and normal gDNA as templates The last sample (with VAF 0) was a normal gDNA control. A linear regression is fitted, with a Pearson correlation coefficient calculated and labeled. (B) Comparison of CNV values obtained for cfDNA samples, extracted from CSF and plasma and tumor gDNA samples (PC gDNA) from PA patients tested in respective multiplexed ddPCR assays. In the test CSF panel, only one sample from patient 58 is higher than the controls.

The availability of tumor gDNA for a majority of *KIAA1549::BRAF* fusion cases in our cohort allowed us to verify a concordance between *BRAF* CNV values and the number of copies of fusion ctDNA. All multiplexed ddPCR assays in our study demonstrated *BRAF* exon 15 duplications on tumor gDNA samples. Calculated *BRAF* CNV values showed a positive correlation with VAF of fusion junction gDNA sequence (*R*^2^ = 0.976, *P* < .0001) (Figure 3A). In more detail, tumor gDNA from patient 3 had a VAF of 0.5 using a patient-specific fusion assay, indicating a monoallelic *BRAF* duplication event in a tumor biopsy sample with 100% purity, while the corresponding screening *BRAF* duplication assay had a CNV = 3.04. We also had a tumor gDNA sample from patient 25 with a low tumor fraction of approximately 20% (fusion VAF—0.1) with CNV—2.22, close to normal control CNV values.

Our calculation of cfDNA concentrations in liquid biopsy samples was based on the concentration of copies of reference exons obtained after ddPCR analysis. The CSF-derived cfDNA concentration was 0.02–34.66 ng/ml with a median of 0.53 ng/ml (mean 4.52 ng/ml). Plasma cfDNA yields were higher: 0.36–13.95 ng/ml with a median of 3.68 ng/ml (mean 4.155 ng/ml) (Figure 2C, Supplementary Table 2).

Additionally, in our assays, we used the set of normal control DNA samples extracted from blood cells (gDNA) and liquid biopsies (CSF and plasma cfDNA). We discovered that *BRAF* CNV ranges can differ depending on the sample material (Figure 3B, Supplementary Figures 3 and 4). As expected for a diploid genome, the median *BRAF* CNV values for normal gDNA cfDNA were ~2.0. For plasma cfDNA samples from PA patients lacking the *BRAF* CNV and expected to display normal CNV values, a median of 2.33 (mean 2.34 ± 0.12) was observed. A similar range for NC plasma cfDNA samples was shown in Ruas et al. publication.^23^

According to our previous validation of the CNV assay, only 6 CSF cfDNA samples had sufficient (>1.9 ng) extracted cfDNA for reliable calculation of *BRAF* CNV. Of those, only patient 58 had *BRAF* exon 15 CNV (=3.42) significantly higher than control values and can be considered positive with the *BRAF* duplication assay.

**Figure 4.**
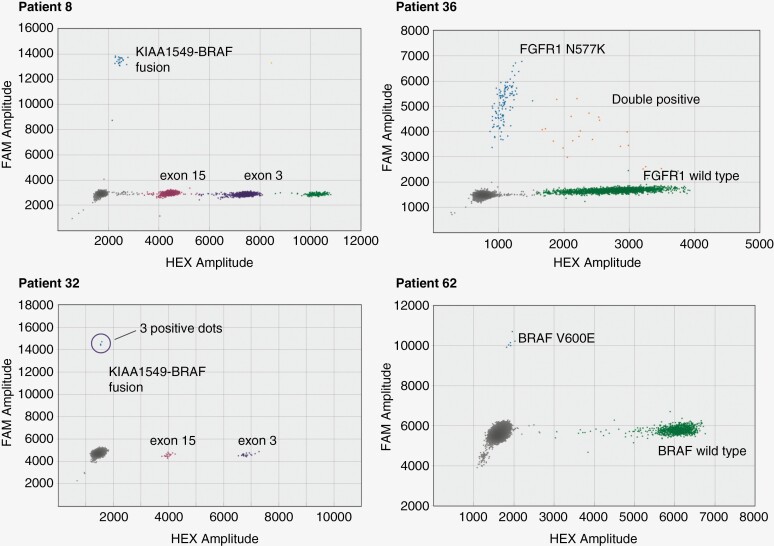
Droplet digital PCR 2D amplitude plots of cfDNA CSF samples with detected ctDNA.

Among all tested CSF samples, fusion breakpoint ctDNA was detected in 4/13 samples (Figure 4, Supplementary Figure 4). Patient 36 had the second highest cfDNA concentration and was positive for mutant *FGFR1* N577K ctDNA with a VAF of 4.42%. Patient 8 also had an elevated amount of cfDNA in CSF compared to the median value of CSF cfDNA yield and had detectable fusion *KIAA1549::BRAF* ctDNA at 0.75% VAF using targeted probes. Patient 62 had a detectable amount of ctDNA *BRAF* V600E with a VAF of 0.66%. The final positive CSF sample from patient 32 had a low concentration of cfDNA in CSF (9 hGEs/ml), and we found the minimal 3 copies of specific *BRAF* fusion ctDNA using 2.6 ml of CSF. All plasma samples were negative for fusion or SNV.

## Discussion

Advances in the molecular characterization of tumors extensively facilitate accurate diagnostic approaches. Information on specific genetic markers enables precise classification and prognosis, guides treatment decisions, and may result in improved patient care and clinical outcomes. Current standard-of-care protocols are based on examining tissue-extracted DNA to perform molecular characterization. Still, ctDNA from liquid biopsies could be an alternative/supplementary source of tumor DNA, especially in pediatric CNS tumor settings where obtaining tissue biopsies can be impossible or lead to significant morbidity.

Pilocytic astrocytoma patients represent a large proportion of pLGG cases and have a well-described genomic landscape with either a *KIAA1549::BRAF* fusion or *BRAF* V600E SNV as the main driver aberrations. In recent years, several groups attempted to use cfDNA extracted from CSF to determine *BRAF* mutational status by NGS. The study by Pan et al. found a *BRAF* fusion in 1 out of 4 PA/PMA CSF samples by applying a custom-designed gene panel and deep sequencing with an average depth of >1500×.^24^ Miller and colleagues detected *KIAA1549::BRAF* fusion in 3 cases of DLGNT using the MSK-IMPACT assay but in 0/4 LGGs.^25^ More recent work shows the possibility of detecting a *BRAF* fusion in 5/10 PA samples using a targeted gene capture method. They developed a protocol that allowed the use of 5 ng of cfDNA for library preparation for custom gene panel sequencing.^26^ Still, even though this amount is relatively low, obtaining similar cfDNA yields from LGGs liquid biopsy samples can be problematic. ddPCR is a targeted method with high sensitivity and specificity that can successfully detect mutant ctDNA copies in low template concentrations.

In our work, we developed a multiplexed ddPCR assay to determine *KIAA1549::BRAF* fusion status by simultaneously detecting patient-specific fusion junction breakpoints and *BRAF* duplication by CNV analysis. The screening with *BRAF* duplication assay was our main interest because it can be applied to all patients without prior knowledge of the genomic sequence of the *BRAF* fusion. To the best of our knowledge, we are presenting here the largest cohort of PA patients for whom CSF and plasma samples have been collected and analyzed. We discovered that most CSF samples had a very low total cfDNA yield; only 6/13 samples had more than 1.9 ng of cfDNA available for analysis. Four of them (4/6) were positive for ctDNA (*KIAA1549::BRAF* fusion detected with either screening or fusion targeted assay, *BRAF* V600E and *FGFR1* N577K). The 2 remaining samples had a large gDNA contamination from blood-mixed CSF or problematic custom ddPCR assay due to the complex intronic DNA sequence around the fusion breakpoint. The final 5th positive result was obtained using a fusion-specific probe on a sample with low cfDNA yield.

We did not find any correlation between tumor size or tumor location and ctDNA detection. We detected the *BRAF* fusion in ctDNA in the CSF sample from the 10-year-old child with the tumor in the 4th ventricle, and since the tumor was located directly in the CSF reservoir, it could be expected to be positive for ctDNA. However, the other 3 cases with tumors located in the 4th ventricle (and available CSF) were negative for ctDNA. We also found *BRAF* fusion breakpoint ctDNA in 1 sample with a low total content of cfDNA. This sample was taken from a 3-year-old patient with a large right cerebellar hemisphere tumor (volume 99 cm^3^). The tumor was not in contact with CSF, but out of 7 CSF samples from patients with similar or larger tumors contacting CSF, only 2 were positive.

The *BRAF* duplication screening assay successfully revealed *BRAF* ctDNA CNV in a single CSF sample from patient 58 with leptomeningeal spread. We assume that the more malignant phenotype characteristic of DLGNT patients compared to PA contributes to a more active release of ctDNA to CSF and is crucial for the successful application of the *BRAF* CNV analysis of cfDNA.

LGGs are characterized by their low malignancy and the fact that they do not shed much cfDNA, making the clinical implementation of screening assays challenging. Thus, most cases have very low cfDNA content in the CSF and a low fraction of tumor-derived cfDNA (VAF for fusion DNA for all positive PA samples was below 20%). So, according to our data, the chances of detecting duplication in cfDNA remain low. In clinical practice, gene panel sequencing of samples with a high total cfDNA amount may be a better alternative since it can detect ctDNA mutated sequences with VAF above 0.5% with high specificity.^27^ Methylation analysis can be applied for samples with low cfDNA (below 5–10 ng) content where successful libraries have been achieved using a low volume of liquid biopsies (below 1 ml of plasma or CSF with at least 1 ng of cfDNA).^28,29^ On the other hand, dPCR analysis of *BRAF* V600 mutation is already slowly entering clinical practice.^30^ Multiplexed *BRAF* mutational status assay is advantageous because it can distinguish between a *BRAF* V600 mutation and *BRAF* fusion, leading to different risk stratification and treatment. The *BRAF* V600 test used in our study already contains BRAF exon 15, and this assay can be easily multiplexed with an additional BRAF exon 3 probe.^31^ Detection of *BRAF* aberrations in CSF can enable access to novel targeted therapies with oral RAF/MEK inhibitors even when tissue biopsies are not available, as in the case of patient 58, who is now eligible for inclusion in 1 arm of the LOGGIC study.^32^

## Conclusions

In this study, we analyzed the mutational status of the liquid biopsy samples from a cohort of pilocytic astrocytoma patients with a ddPCR approach. We conclude that analysis of *BRAF* duplication alone had limited clinical utility with a high risk of false negatives. However, if a *BRAF* duplication is detected, it may have a clinical impact on prognosis and treatment, and therefore, CSF analysis in pLGG may still be a viable alternative if no tissue biopsy is available. We successfully show that the assay can be multiplexed with additional probes and thus combining the *BRAF* fusion assay with a *BRAF* V600 screening ddPCR test will provide greater clinical utility. Our study emphasizes the difficulties in CSF cfDNA analysis and prompts us to improve current diagnostic approaches, especially in LGGs.

## Supplementary Material

vdae008_suppl_Supplementary_Tables_1

vdae008_suppl_Supplementary_Figures_1

vdae008_suppl_Supplementary_Figures_2

vdae008_suppl_Supplementary_Tables_2

vdae008_suppl_Supplementary_Figures_3

vdae008_suppl_Supplementary_Figures_4
