# Supplementary material for: The potential of liquid biopsy for detection of the KIAA1549-BRAF fusion in circulating tumor DNA from children with pilocytic astrocytoma
Source: Neurooncol Adv. 2024 Jan 24;6(1):vdae008. doi: 10.1093/noajnl/vdae008 (PMC10874216; doi:10.1093/noajnl/vdae008)
Supplement: vdae008_suppl_Supplementary_Tables_1 [file vdae008_suppl_supplementary_tables_1.docx]

**The potential of liquid biopsy for detection of the KIAA1549-BRAF fusion in circulating tumor DNA from children with pilocytic astrocytoma.**

Supplementary table 1. List of ddPCR assays.

| UPN | Genomic aberration |  | Sequence | Amplicon | False positives (fusion) | Supplier |
| --- | --- | --- | --- | --- | --- | --- |
| 10 | *KIAA1549::BRAF* | Forward Primer | GAGAGTAGCGATAACACTGA | 91 | 0 | IDT |
|  |  | Probe | AAGAGGAAGAATCAGCGAGCC |  |  |  |
|  |  | Reverse Primer | ACTCCAGCTAGTGCAATG |  |  |  |
| 12 | *KIAA1549::BRAF* | Forward Primer | GACAGTCGCTACTCAACA | 73 | 0 | IDT |
|  |  | Probe | TTTACCTATCAGCTGCCATGC |  |  |  |
|  |  | Reverse Primer | CAGTTCTGATGCTGAACAC |  |  |  |
| 3 | *KIAA1549::BRAF* | Forward Primer | GTGATATGCACAAAGGACTAG | 107 | 0 | IDT |
|  |  | Probe | CGTGGCCTGACTCACTGA |  |  |  |
|  |  | Reverse Primer | CGAGGGTAGGAGTGTTATG |  |  |  |
| 8 | *KIAA1549::BRAF* | Forward Primer | TGGCTCAAGCCTGGATTT | 76 | 0 | IDT |
|  |  | Probe | ATGTCCCAGCTCATGCAGTC |  |  |  |
|  |  | Reverse Primer | GCTTCAAGGATGGGAGAAG |  |  |  |
| 17 | *KIAA1549::BRAF* | Forward Primer | CCAACTGCTACTGGATTTCT | 115 | 1 | IDT |
|  |  | Probe | CTGAAAGTCCCACAGGCATCTT |  |  |  |
|  |  | Reverse Primer | ACAGTTGGTGGAAACTGCATA |  |  |  |
| 25 | *KIAA1549::BRAF* | Forward Primer | GTGATGTCATTAACTTTCCAAC | 105 | 0 | IDT |
|  |  | Probe | CAGGCAGATCACGAGGTCAG |  |  |  |
|  |  | Reverse Primer | GGTTTCACCATGTAGCCA |  |  |  |
| 11 | *KIAA1549::BRAF* | Forward Primer | GTGGCACATGTTGTTCTG | 87 | 0 | IDT |
|  |  | Probe | CCGTTTACTGGTCCCCAGGAG |  |  |  |
|  |  | Reverse Primer | GGGCACCTTATGGAACTTA |  |  |  |
| 30 | *KIAA1549::BRAF* | Forward Primer | CAGAAACCTGGCATTATCC | 103 | 0 | IDT |
|  |  | Probe | TCACTCCTCTCCTCTACTACAAA |  |  |  |
|  |  | Reverse Primer | AAGGAGGAGGGCAAGATT |  |  |  |
| 32 | *KIAA1549::BRAF* | Forward Primer | AGTCTTCAACATTTTCTACAGA | 105 | 0 | IDT |
|  |  | Probe | CCAGCCTCTGTTCCTCCATCT |  |  |  |
|  |  | Reverse Primer | GAAATTAAAGTGTATGGGACAGG |  |  |  |
| 33 | *KIAA1549::BRAF* | Forward Primer | CAAAGTGGCTCATAGAAAGG | 94 | 0 | IDT |
|  |  | Probe | AGACTCAGTTGCTCTGTCGC |  |  |  |
|  |  | Reverse Primer | GTCGGCTCATGTAATTATGG |  |  |  |
| 45 | *KIAA1549::BRAF* | Forward Primer | CAACCCTAACCTGGACATA | 117 | 0 | IDT |
|  |  | Probe | GGTCTTGCTAGATAGAGGTGAACC |  |  |  |
|  |  | Reverse Primer | GTATGTCTTCCAGAGAGCAA |  |  |  |
| 46 | *KIAA1549::BRAF* | Forward Primer | GCTGCTCAATTGATTGCC | 112 | 0 | IDT |
|  |  | Probe | AGTAACAACACGAACCTGCCA |  |  |  |
|  |  | Reverse Primer | CAGTGTGTAGCGTGATTCT |  |  |  |
| 36 | *FGFR1* N577K |  | dHsaMDS559561726 |  | 0 | Bio-Rad |
| 62 | *BRAF V600* |  | *BRAF* V600 Screening Kit (#12001037) |  | 0 | Bio-Rad |
| 7 | *KIF21B::NTRK1* | Forward Primer | TCTCGAACTGCAGAACTC | 92 | 0 | IDT |
|  |  | Probe | CCAATGGCCTCATTGCCATC |  |  |  |
|  |  | Reverse Primer | ATCTCCCGGATGTAGTTC |  |  |  |
|  | *NOS1AP::KIF21B* | Forward Primer | CTCAGCTCCAGAGACCAG | 86 | 1 | IDT |
|  |  | Probe | CCTCCACCTTGGGCTACTCA |  |  |  |
|  |  | Reverse Primer | CCTTCTAGTGATGGGCATTC |  |  |  |
|  | *BRAF* exon 3 | Forward Primer | CAAGTCACCACAAAAACCTATC | 98 |  | IDT |
|  |  | Probe | AGTCTTCCTGCCCAACAAACAG |  |  |  |
|  |  | Reverse Primer | ACAAAGAAACAGCAAAATGGTG |  |  |  |
|  | *BRAF* exon 15 | Forward Primer | CATGAAGACCTCACAGTAA | 95 |  | IDT |
|  |  | Probe | CAGTGAAATCTCGATGGAGTGG |  |  |  |
|  |  | Reverse Primer | GATCCAGACAACTGTTCAAA |  |  |  |
