# Supplementary material for: The potential of liquid biopsy for detection of the KIAA1549-BRAF fusion in circulating tumor DNA from children with pilocytic astrocytoma
Source: Neurooncol Adv. 2024 Jan 24;6(1):vdae008. doi: 10.1093/noajnl/vdae008 (PMC10874216; doi:10.1093/noajnl/vdae008)

**The potential of liquid biopsy for detection of the KIAA1549-BRAF fusion in circulating tumor DNA from children with pilocytic astrocytoma.**

Supplementary figure 1. Chromosomal copy number variation profiles detected in pilocytic astrocytoma patients and generated with ASCAT package.

Patient 11
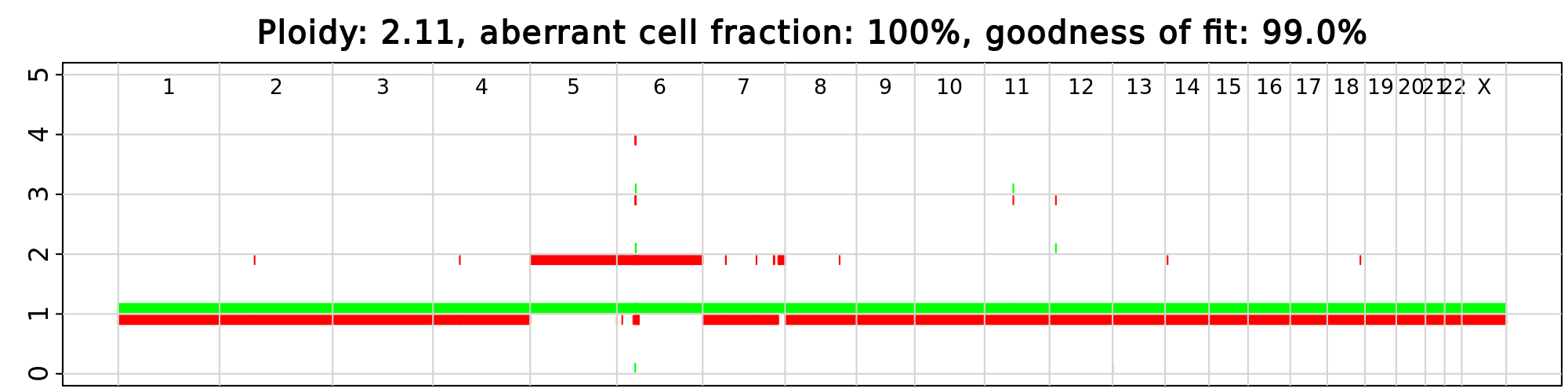


Patient 30


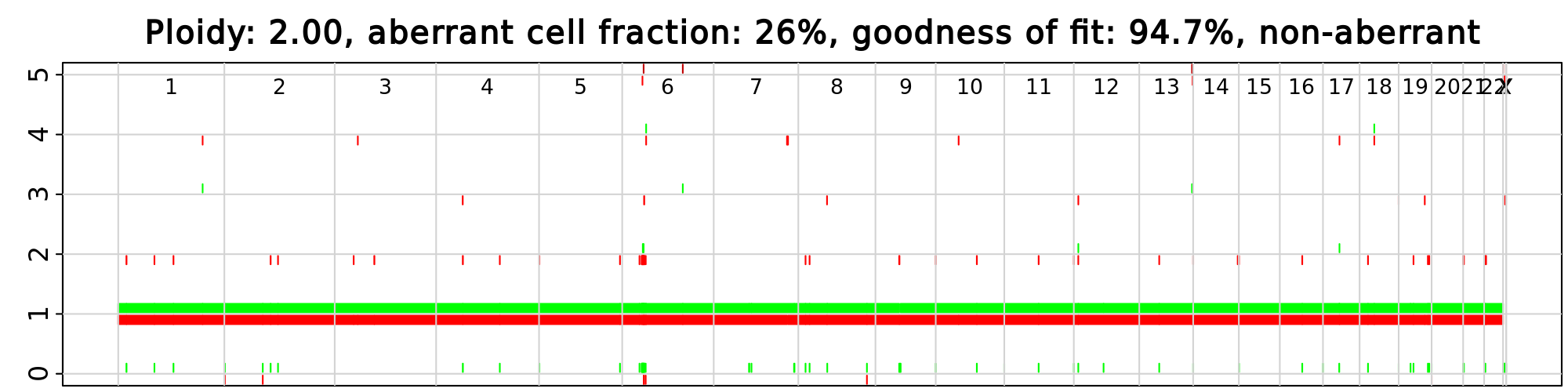


Patient 32


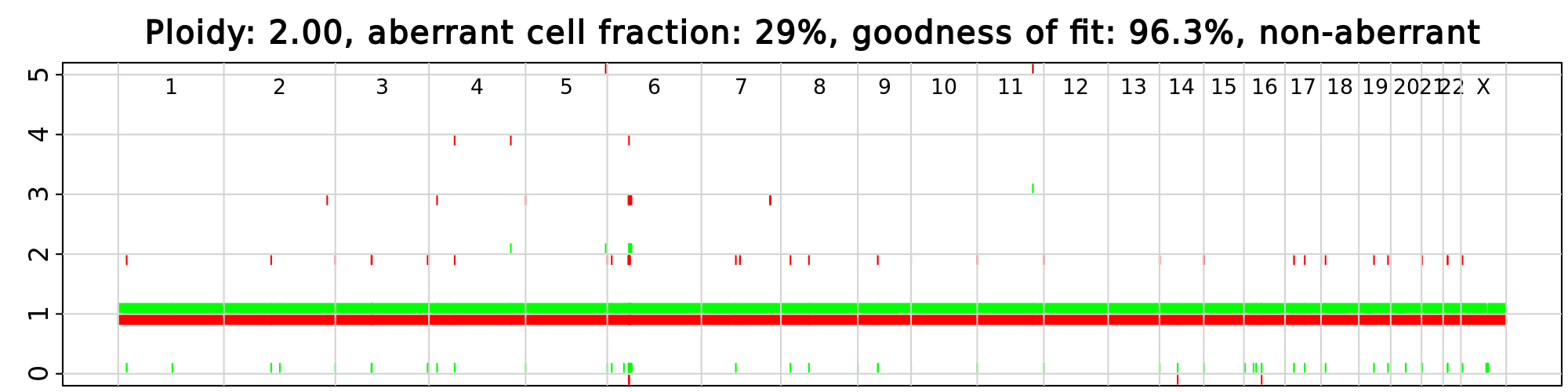


Patient 33


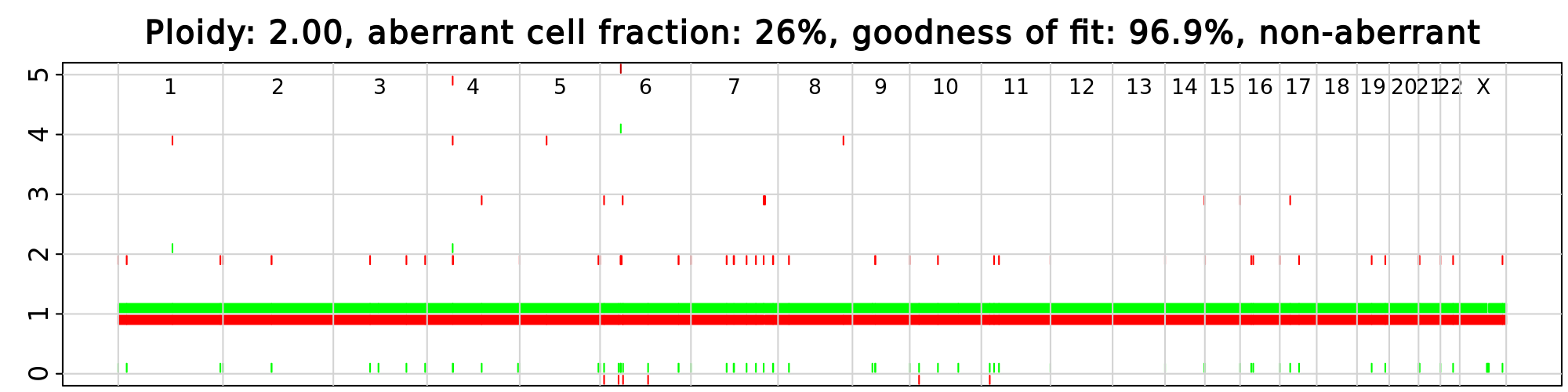


Patient 45
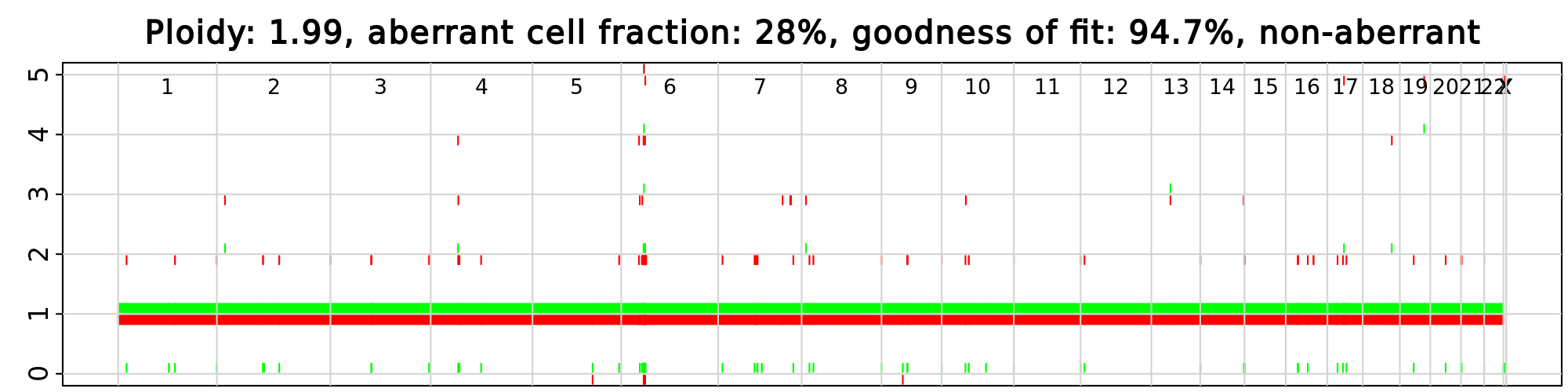


Patient 46


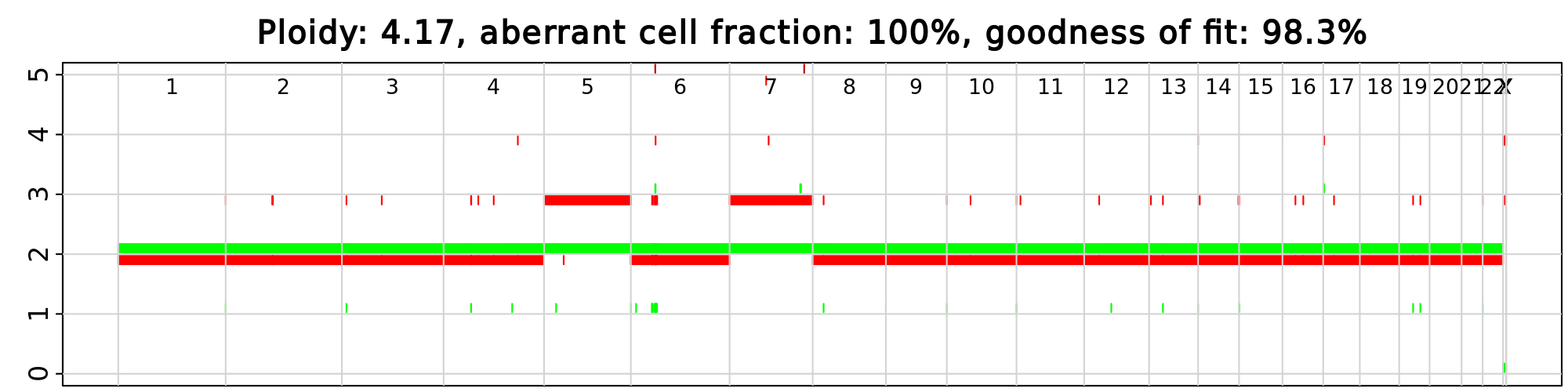


Patient 12


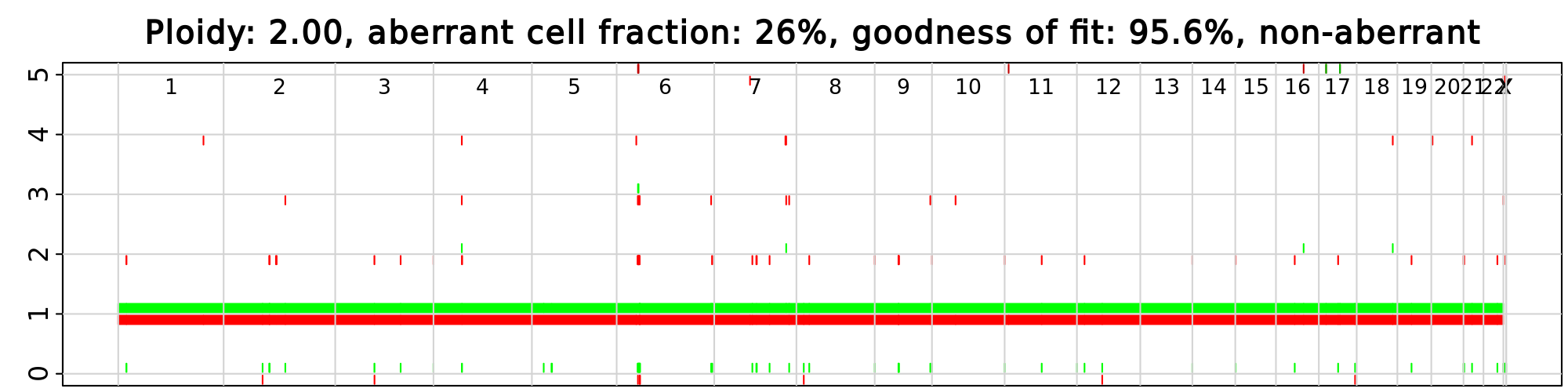


Patient 36


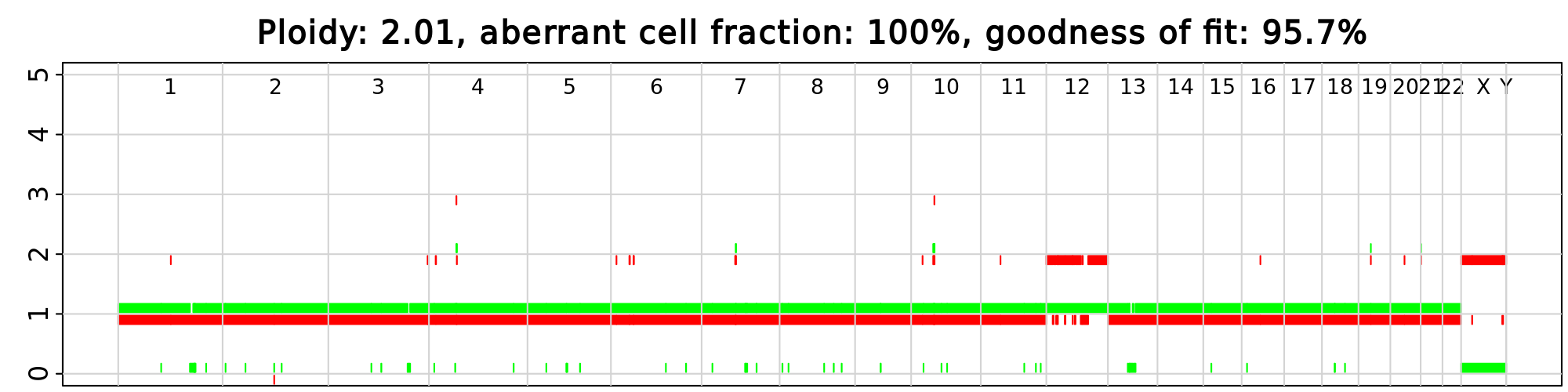


Patient 3


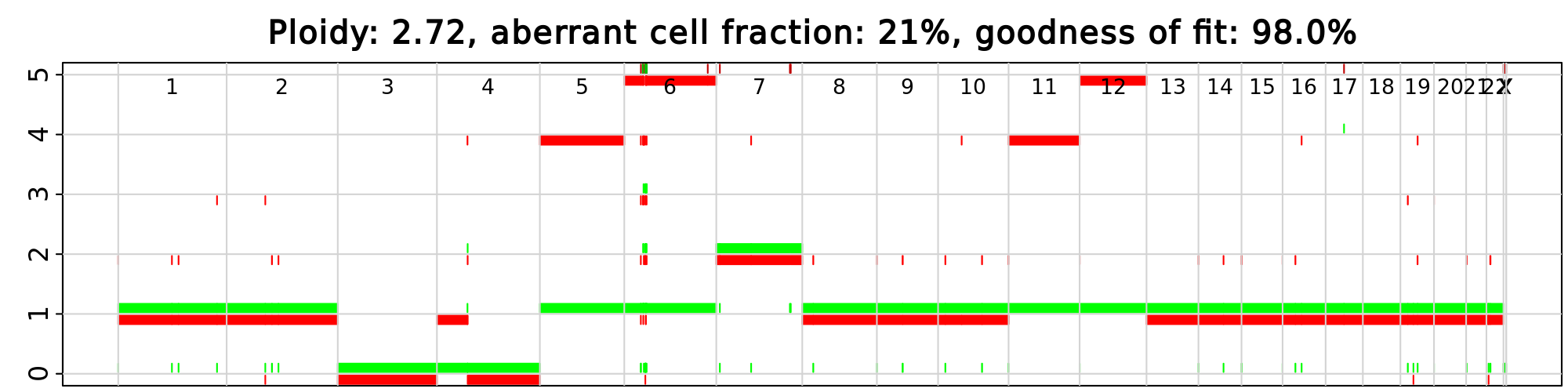


Patient 7
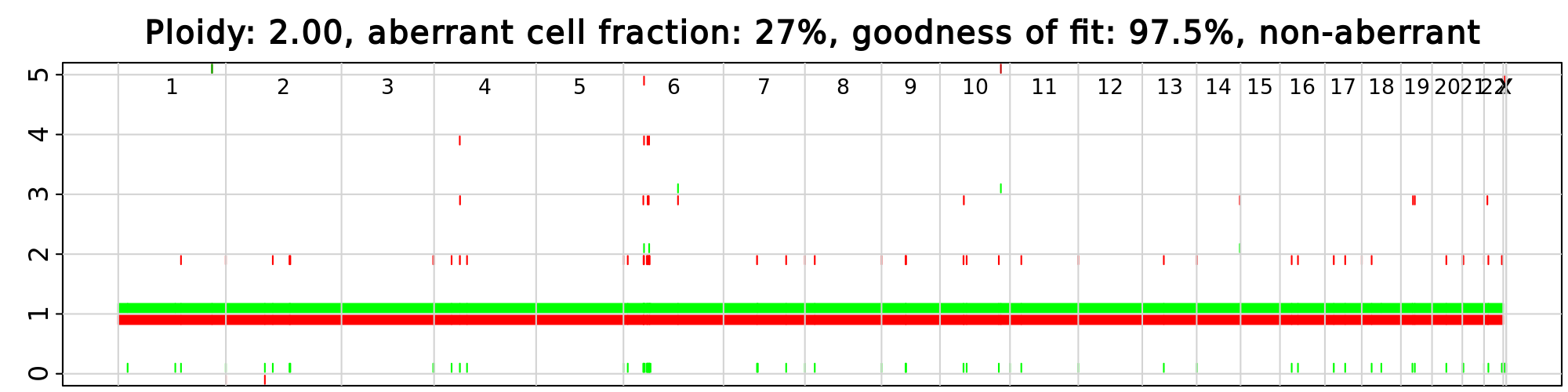


Patient 8
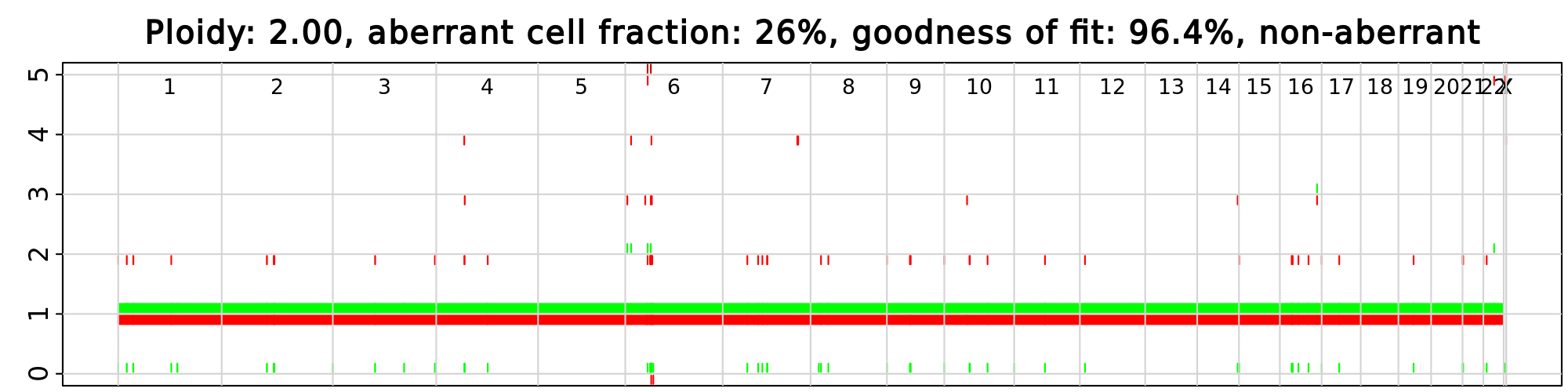


Patient 17 (primary)


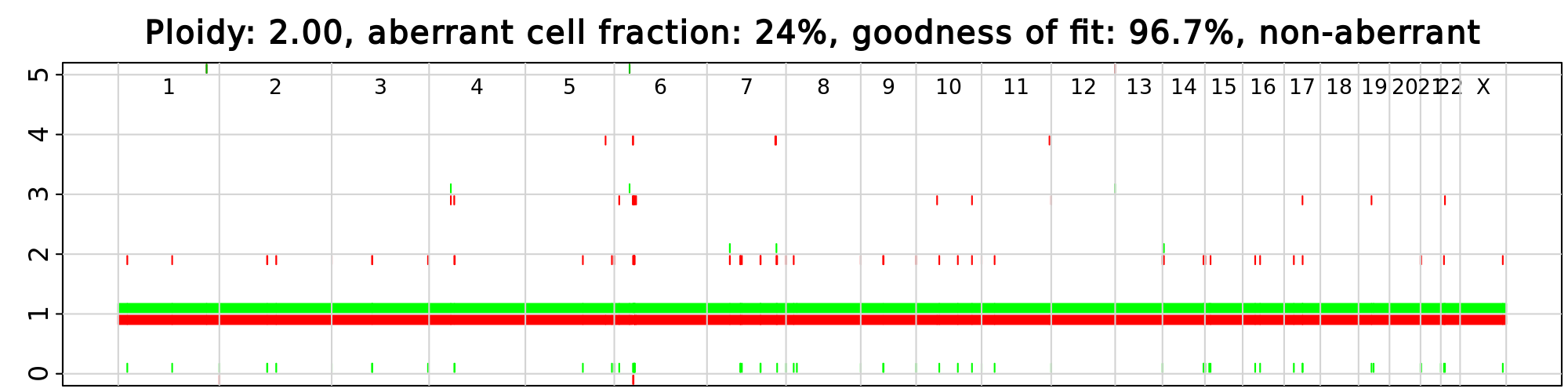


Patient 17 (relapse)
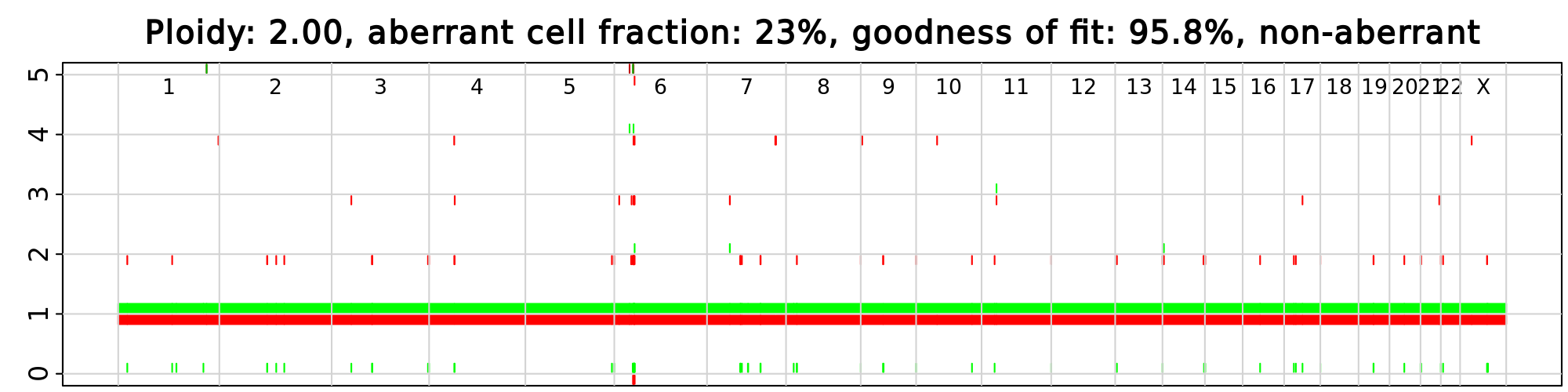


Patient 25


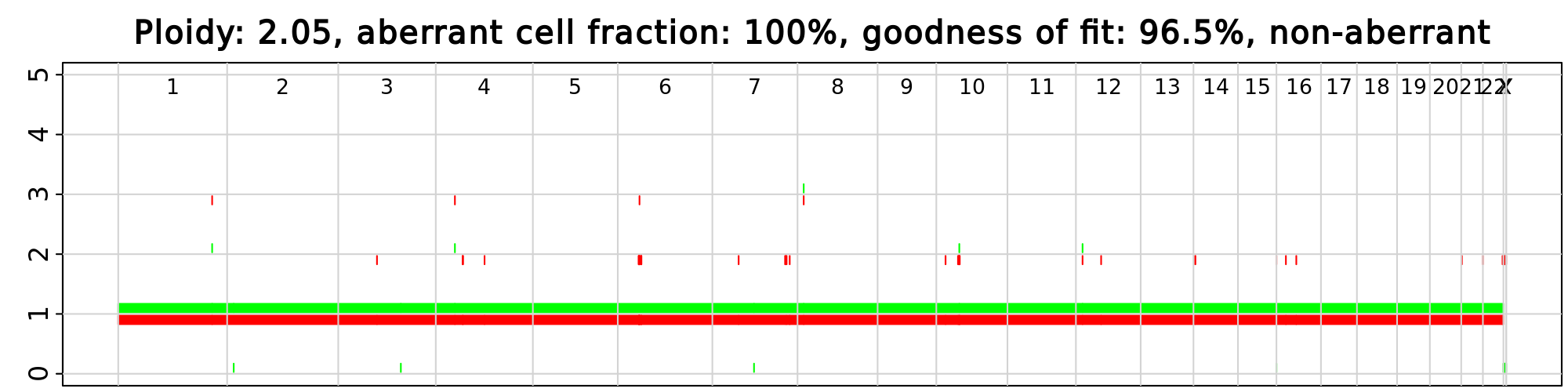


Patient 10


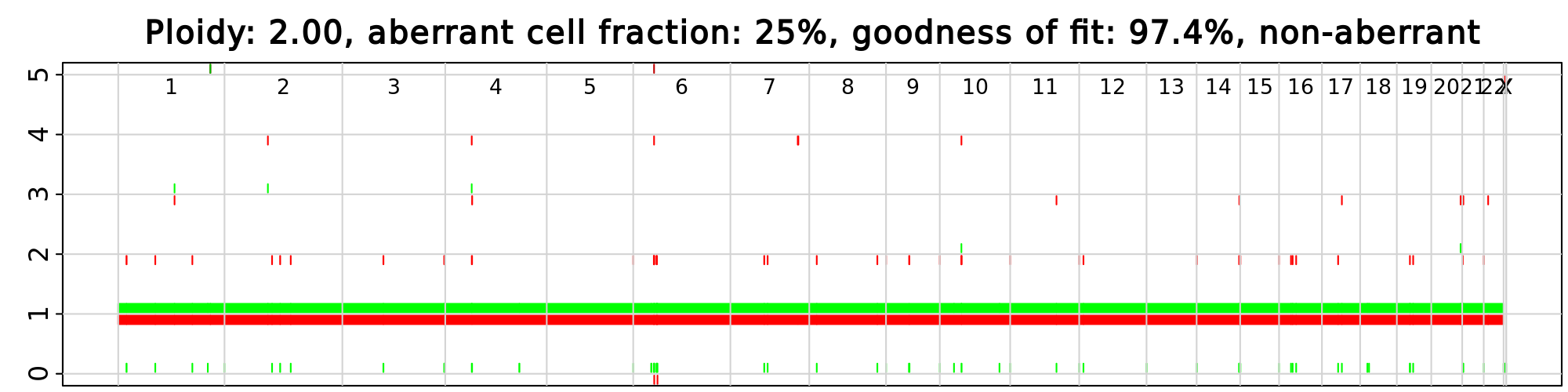

Supplement: vdae008_suppl_Supplementary_Figures_1 [file vdae008_suppl_supplementary_figures_1.docx]
