# Supplementary material for: The potential of liquid biopsy for detection of the KIAA1549-BRAF fusion in circulating tumor DNA from children with pilocytic astrocytoma
Source: Neurooncol Adv. 2024 Jan 24;6(1):vdae008. doi: 10.1093/noajnl/vdae008 (PMC10874216; doi:10.1093/noajnl/vdae008)
Supplement: vdae008_suppl_Supplementary_Figures_2 [file vdae008_suppl_supplementary_figures_2.docx]

**The potential of liquid biopsy for detection of the KIAA1549-BRAF fusion in circulating tumor DNA from children with pilocytic astrocytoma.**

Supplementary figure 2.


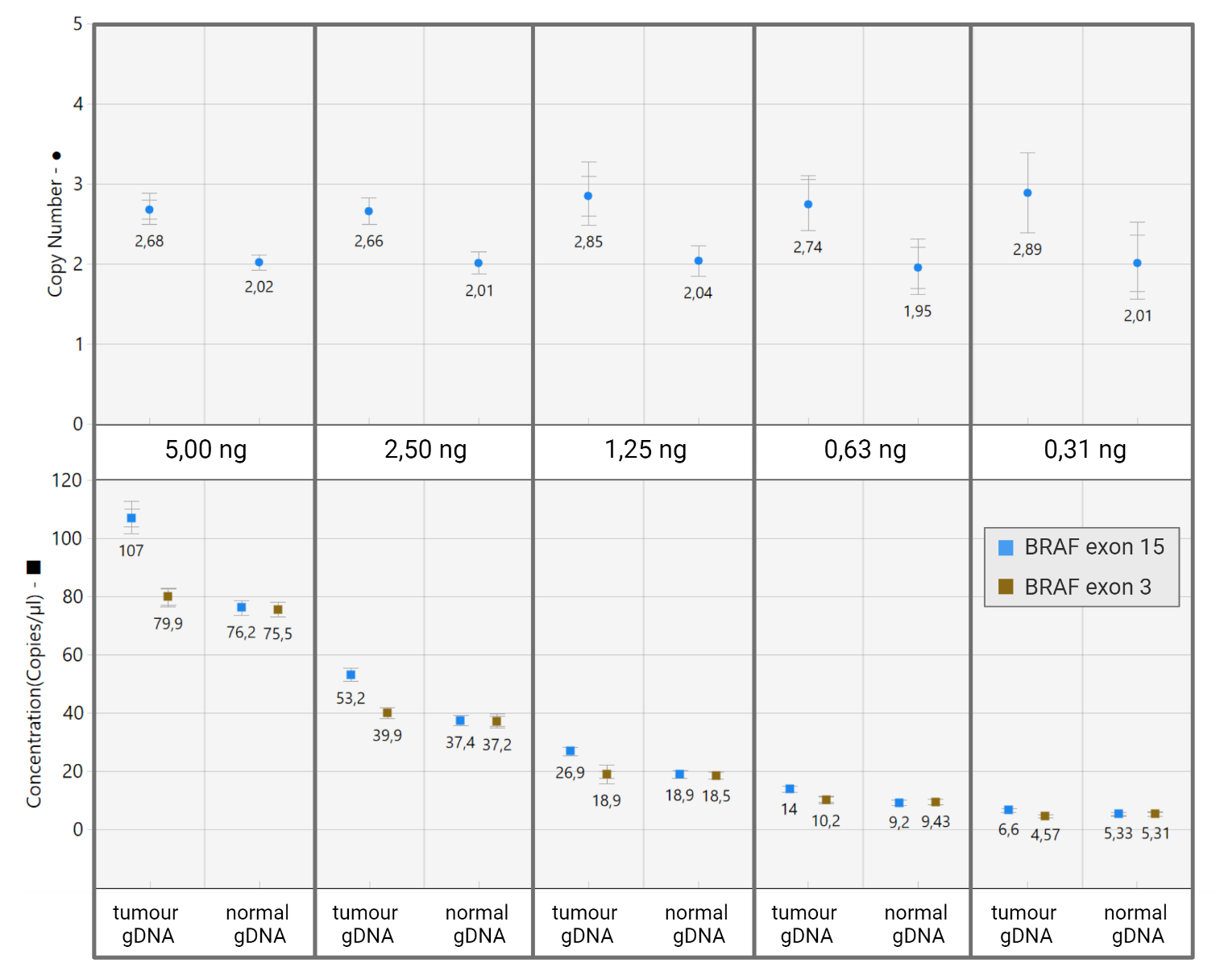


Evaluation of limits of detection of *BRAF* exon15 duplication in multiplexed ddPCR assay. We performed a series of dilutions of gDNA extracted from blood cells (normal gDNA) and a tumor biopsy sample from patient 11 (tumor gDNA) starting from 5 ng input per well (all wells in triplicate). The bottom part shows the average number of copies of *BRAF* exon 3 and exon 15 per uL of reaction mix detected in multiplexed ddPCR assay. The top part represents calculated copy number variation values for each input gDNA concentration. The lowest concentration where we did not see overlapping error bars is 0.63 ng, which confirms that assay can be used with low input of DNA (down to 1.9 ng in total) for samples with significant tumor fraction.
