# Supplementary material for: The potential of liquid biopsy for detection of the KIAA1549-BRAF fusion in circulating tumor DNA from children with pilocytic astrocytoma
Source: Neurooncol Adv. 2024 Jan 24;6(1):vdae008. doi: 10.1093/noajnl/vdae008 (PMC10874216; doi:10.1093/noajnl/vdae008)
Supplement: vdae008_suppl_Supplementary_Tables_2 [file vdae008_suppl_supplementary_tables_2.docx]

**The potential of liquid biopsy for detection of the KIAA1549-BRAF fusion in circulating tumor DNA from children with pilocytic astrocytoma.**

Supplementary table 2. Summary of ctDNA results.

| patient | genetic aberration | volume CSF, ml | volume plasma, ml | CSF ex3 copies | plasma ex3 copies | ng from copies of ex3 (CSF) | ng from copies of ex3 (plasma) | concentration (CSF) | concentration(plasma) |
| --- | --- | --- | --- | --- | --- | --- | --- | --- | --- |
| 11 | *KIAA1549::BRAF* | 0 | 2 |  | 1887 |  | 7.55 |  | 3.77 |
| 30 | *KIAA1549::BRAF* | 4 | 3 | 99.8 | 10460 | 0.40 | 41.84 | 0.100 | 13.95 |
| 32 | *KIAA1549::BRAF* | 2.6 | 3 | 22.29 | 268 | 0.09 | 1.07 | 0.034 | 0.36 |
| 33 | *KIAA1549::BRAF* | 4 | 3 | 30.67 | 804 | 0.12 | 3.22 | 0.031 | 1.07 |
| 10 | *KIAA1549::BRAF* | 6 | 2 | 170 | 2280 | 0.68 | 9.12 | 0.113 | 4.56 |
| 45 | *KIAA1549::BRAF* | 4 | 4 | 295.7 | 2954 | 1.18 | 11.82 | 0.296 | 2.95 |
| 46 | *KIAA1549::BRAF* | 4 | 4 | 34679 | 4491 | 138.72 | 17.96 | 34.679 | 4.49 |
| 12 | *KIAA1549::BRAF* | 0 | 3 |  | 6317 |  | 25.27 |  | 8.42 |
| 36 | *FGFR1* N577K | 1.2 | 4 | 3475 | 6473 | 13.90 | 25.89 | 11.583 | 6.47 |
| 3 | *KIAA1549::BRAF* | 5 | 3 | 7560 | 549 | 30.24 | 2.20 | 6.048 | 0.73 |
| 7 | *KIF21B::NTRK1*. *NOS1AP::KIF21B* | 1 | 2.5 | 189.9 | 1897 | 0.76 | 7.59 | 0.760 | 3.04 |
| 8 | *KIAA1549::BRAF* | 4 | 4 | 3790 | 3684 | 15.16 | 14.74 | 3.790 | 3.68 |
| 17 | *KIAA1549::BRAF* | 3 | 2 | 14.92 | 821 | 0.06 | 3.28 | 0.020 | 1.64 |
| 25 | *KIAA1549::BRAF* | 0 | 3 |  | 1854 |  | 7.41 |  | 2.47 |
| 58 | *KIAA1549::BRAF* | 3.65 | 0 | 482.75 |  | 1.93 |  | 0.529 |  |
| 62 | *BRAF* V600E | 6 | 3 | 1081.51 | 3537.25 | 4.33 | 14.15 | 0.72 | 4.72 |
