# Supplementary material for: The potential of liquid biopsy for detection of the KIAA1549-BRAF fusion in circulating tumor DNA from children with pilocytic astrocytoma
Source: Neurooncol Adv. 2024 Jan 24;6(1):vdae008. doi: 10.1093/noajnl/vdae008 (PMC10874216; doi:10.1093/noajnl/vdae008)
Supplement: vdae008_suppl_Supplementary_Figures_3 [file vdae008_suppl_supplementary_figures_3.docx]

**The potential of liquid biopsy for detection of the KIAA1549-BRAF fusion in circulating tumor DNA from children with pilocytic astrocytoma.**

Supplementary figure 3.

Comparison of CNV values obtained for tumor gDNA samples (PC gDNA), CSF and plasma cfDNA from PA patients and a set of normal controls that included NC gDNA (from a single batch), cfDNA from plasma (NC plasma) extracted from several different batches of plasma and cfDNA from CSF (NC CSF) obtained from 2 patients with non-malignant neurological conditions. Everything was tested with respective multiplexed ddPCR assays. In the NС CSF column, all clustered CNVs were obtained from a single CSF sample with very high DNA concentration, probably due to a large portion of gDNA. The outlier CNV dot (= 2.66) was from the second sample with a low cfDNA amount. In the PС gDNA column, the lowest dot represents a tumor biopsy sample with a low tumor cell fraction in it.
