# Supplementary material for: The potential of liquid biopsy for detection of the KIAA1549-BRAF fusion in circulating tumor DNA from children with pilocytic astrocytoma
Source: Neurooncol Adv. 2024 Jan 24;6(1):vdae008. doi: 10.1093/noajnl/vdae008 (PMC10874216; doi:10.1093/noajnl/vdae008)

**The potential of liquid biopsy for detection of the KIAA1549-BRAF fusion in circulating tumor DNA from children with pilocytic astrocytoma.**

Supplementary figure 4. Detailed results of ddPCR tests of all liquid biopsy samples from pilocytic astrocytoma patients.


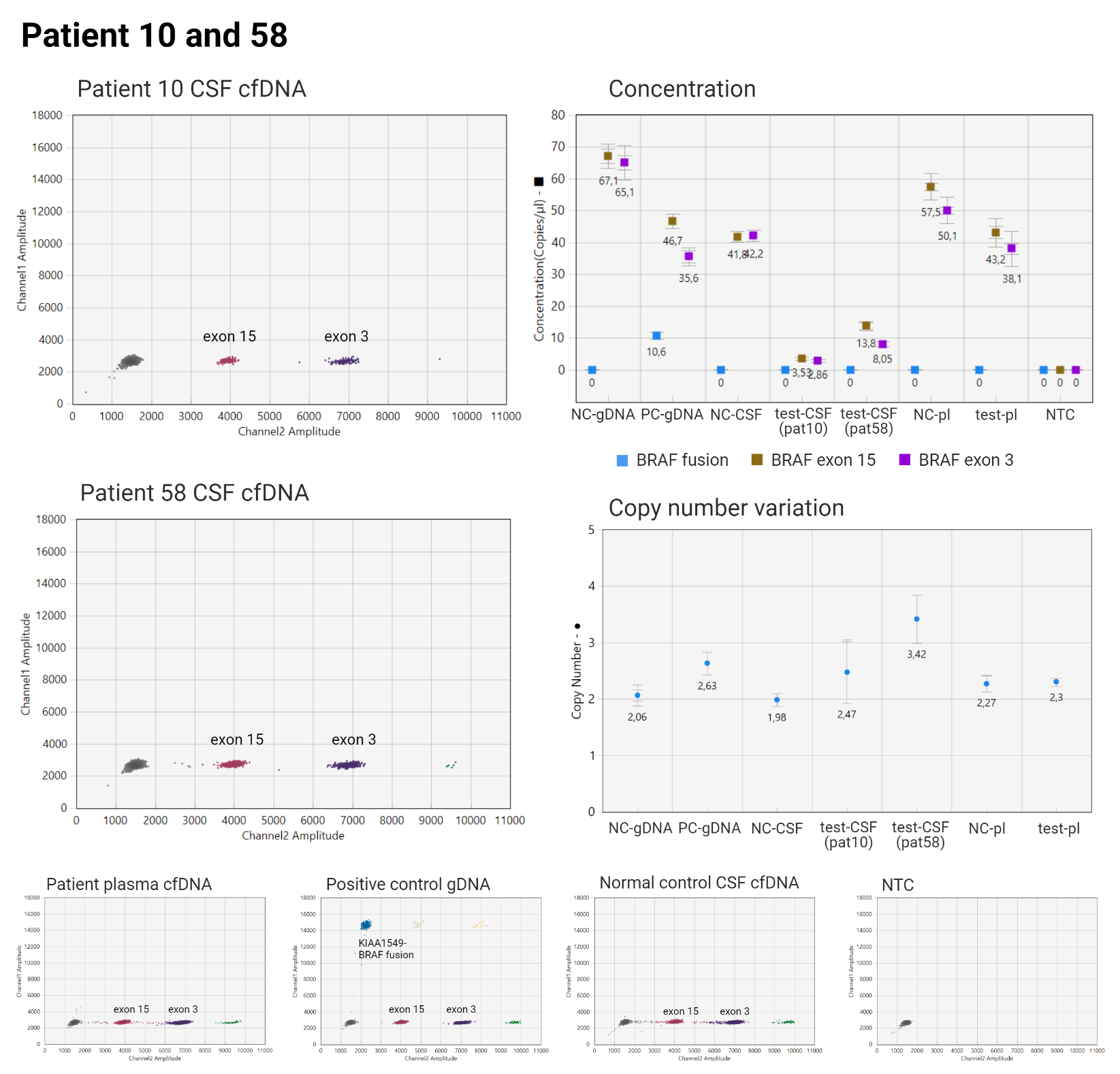


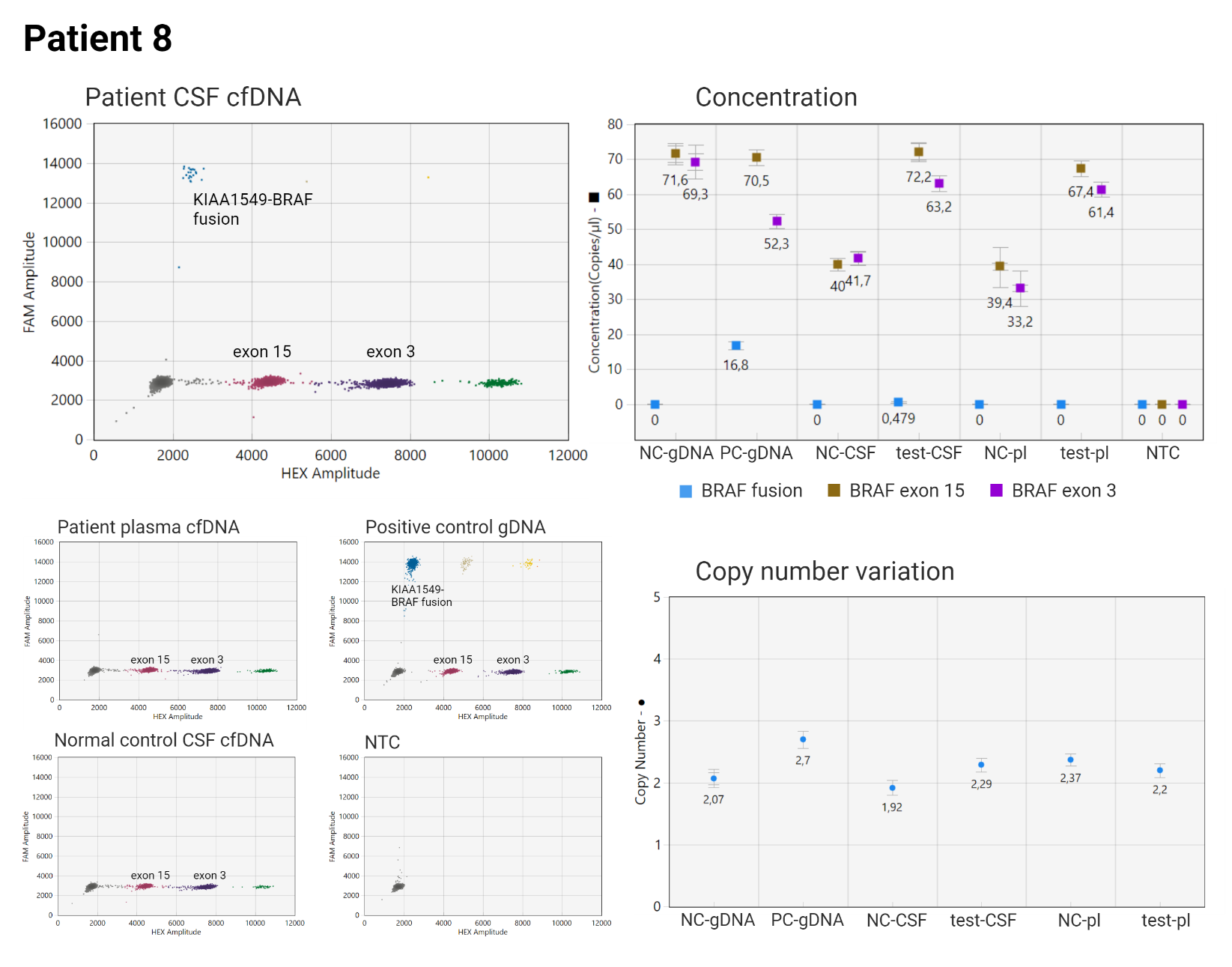


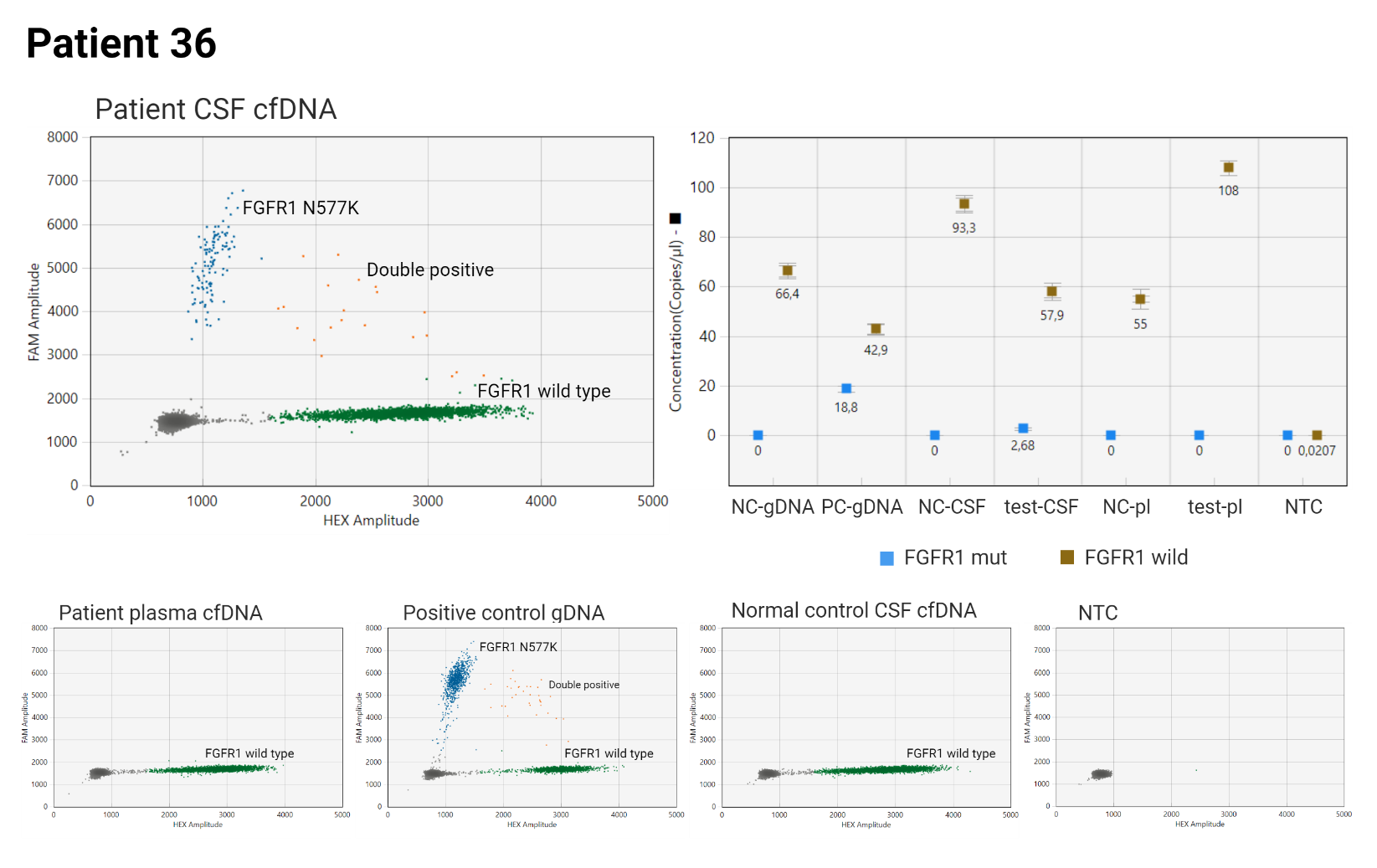


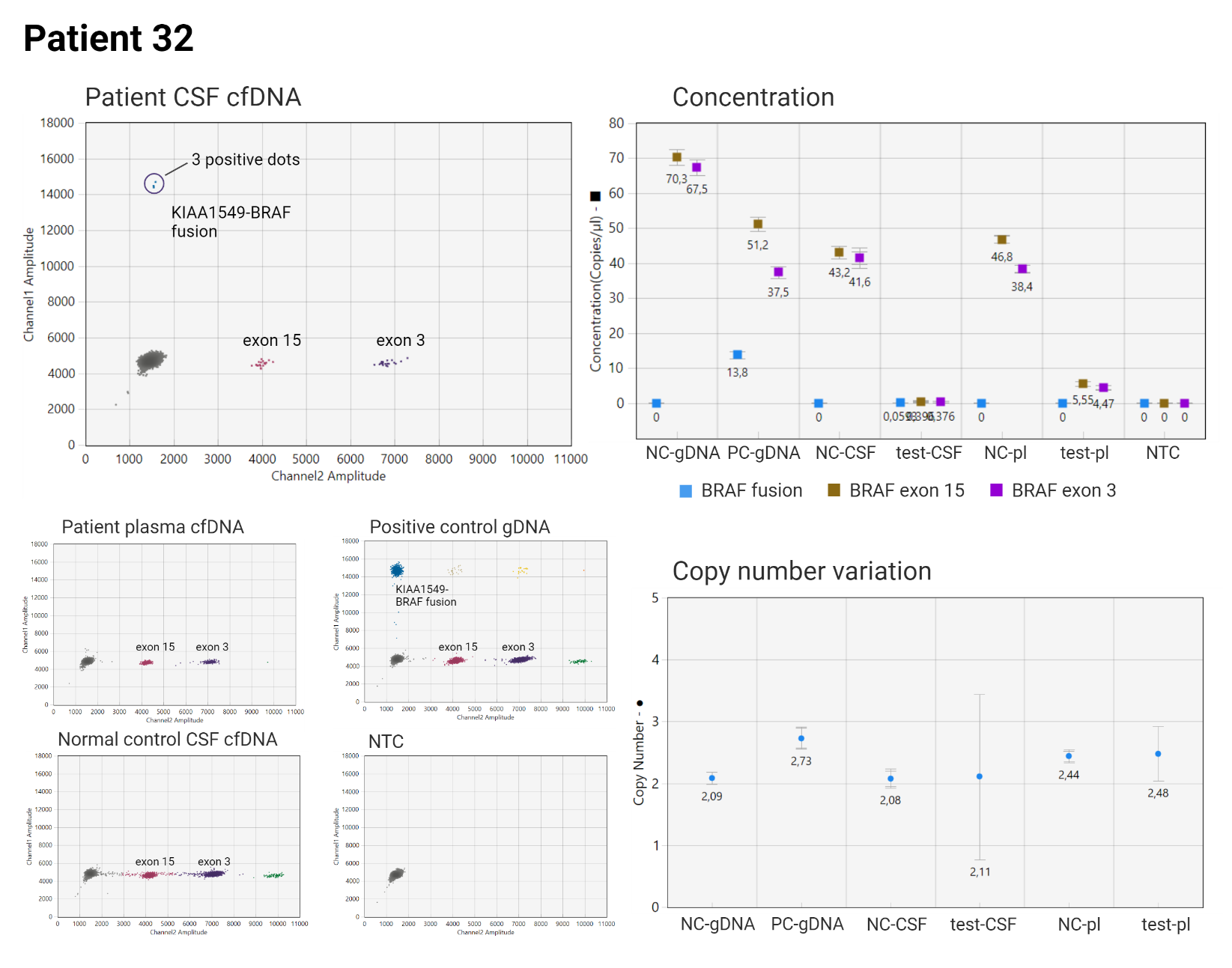


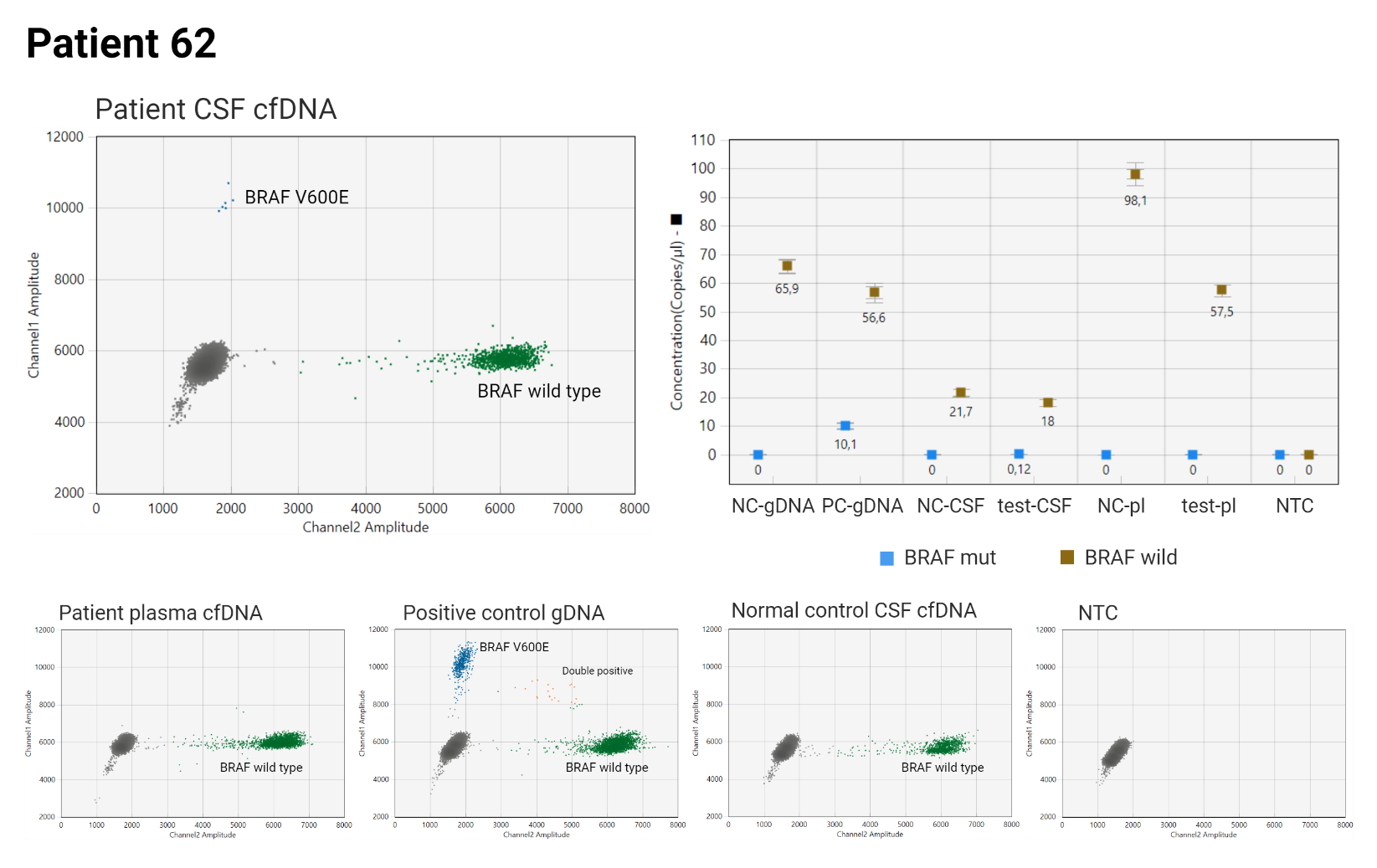


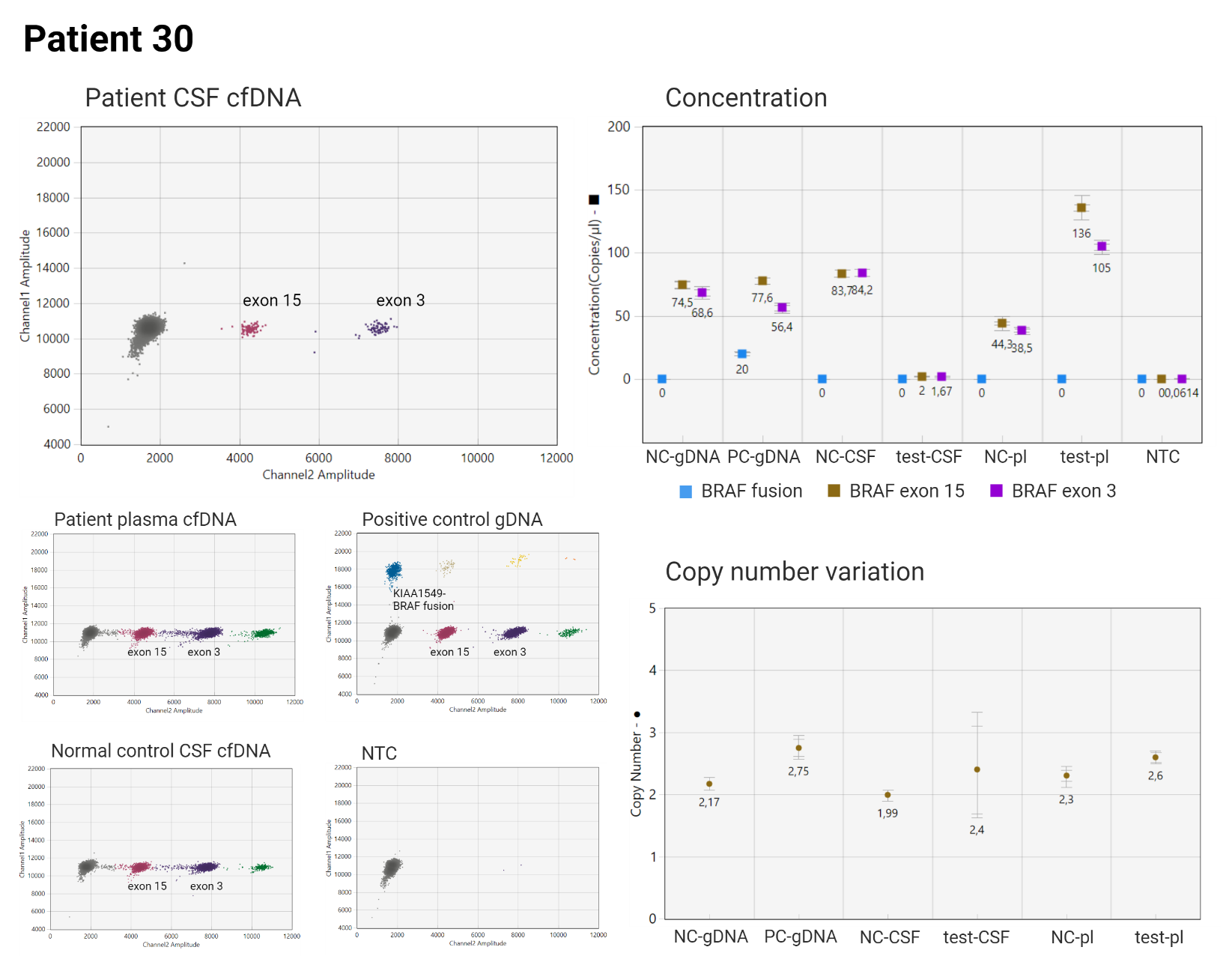


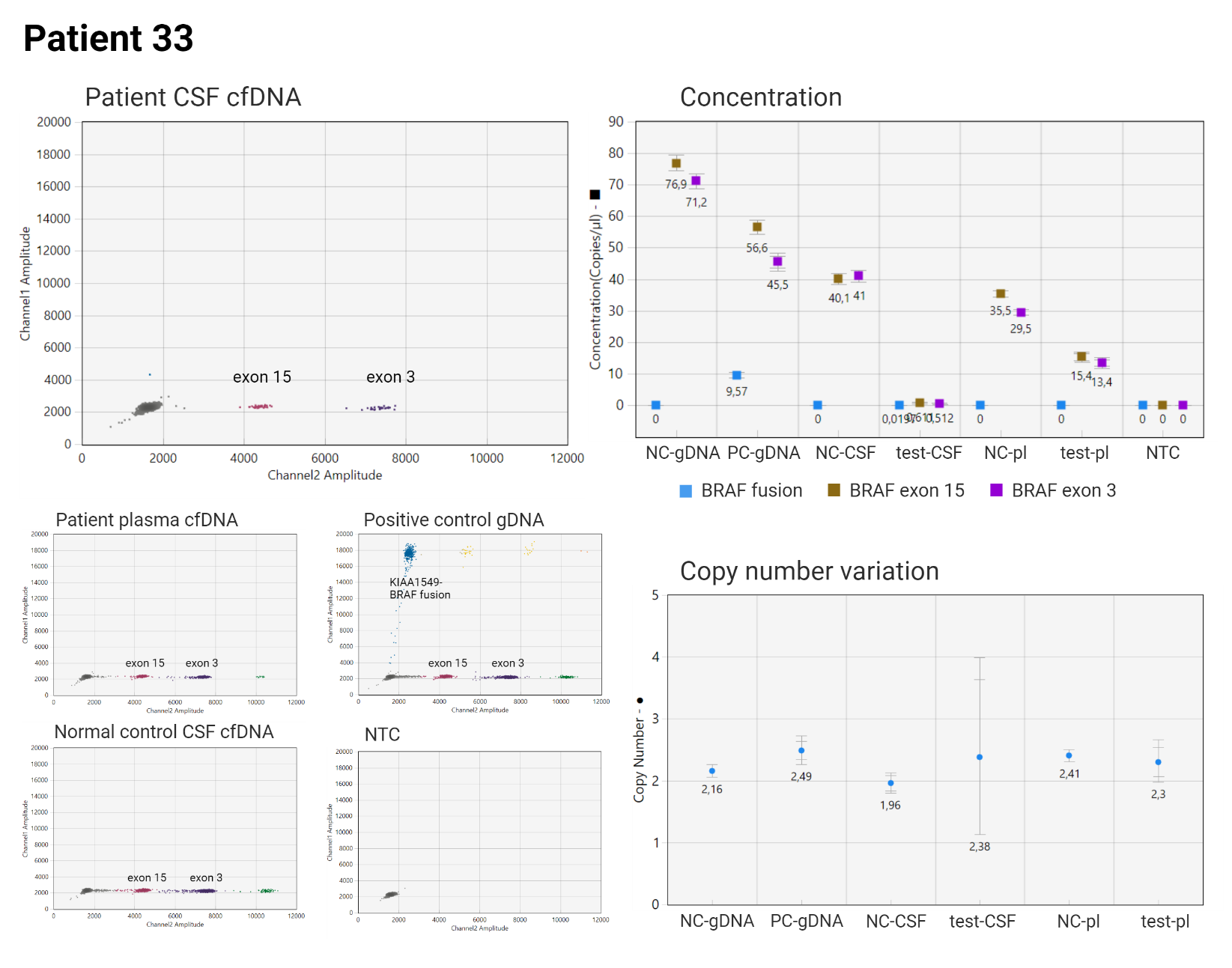


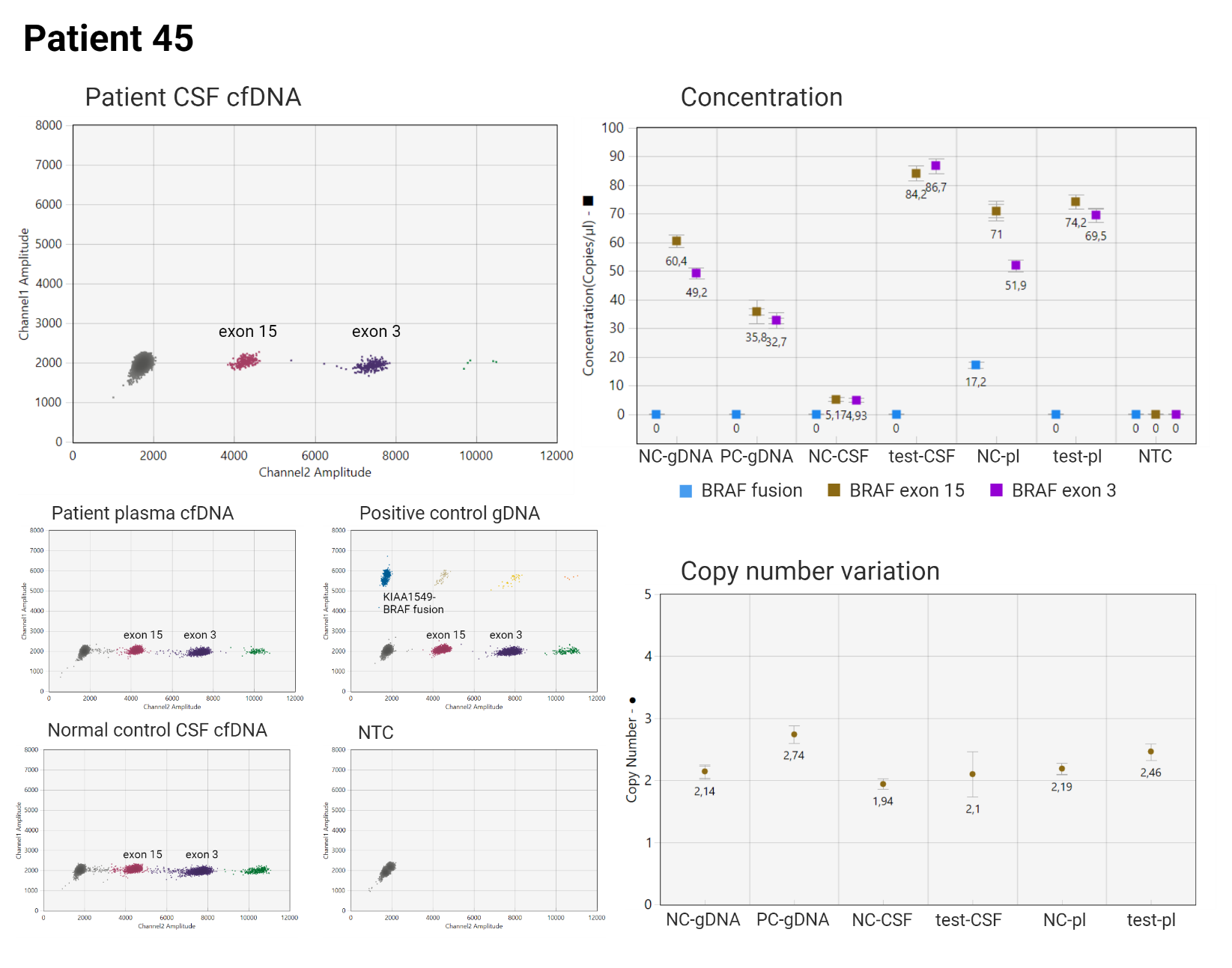


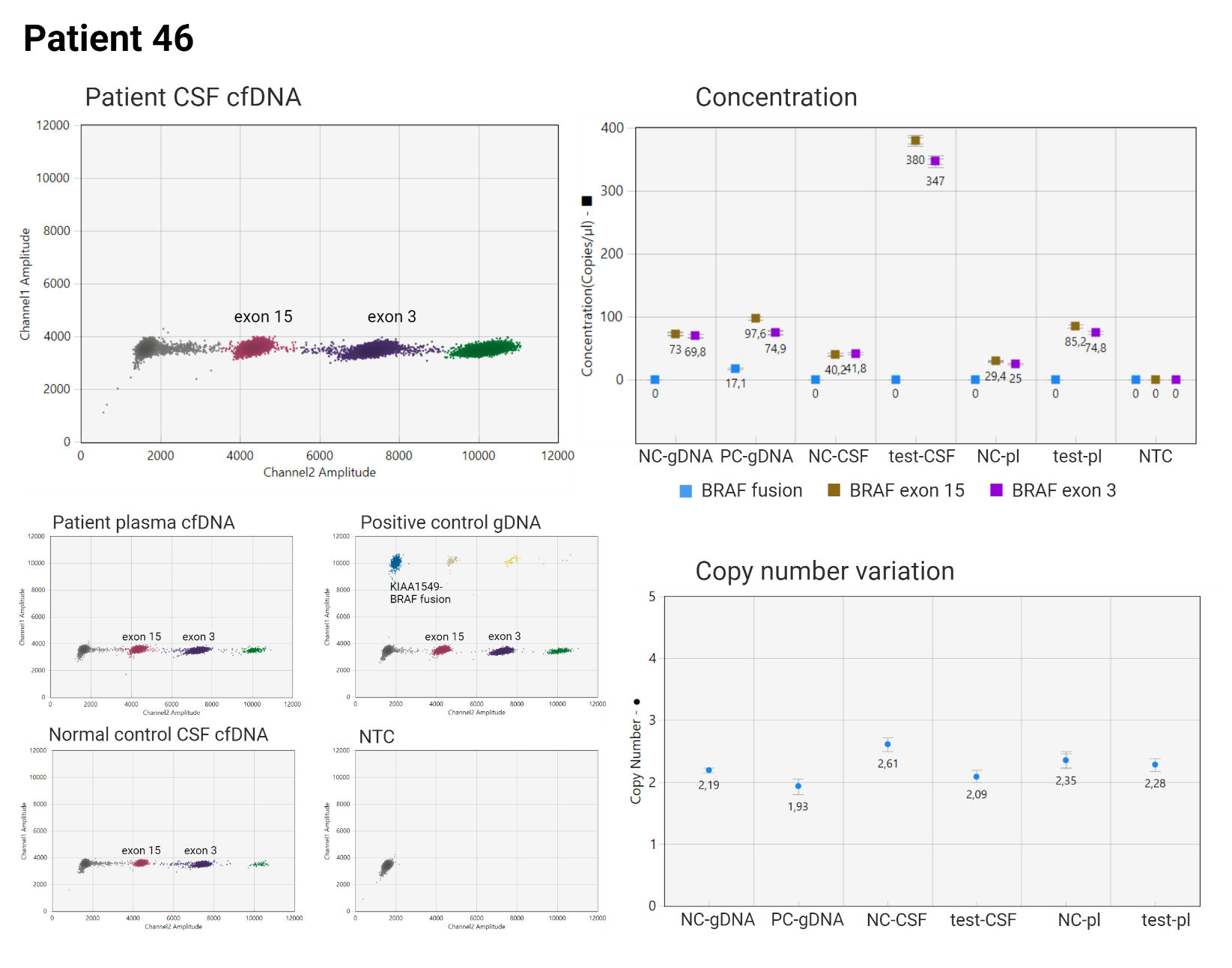

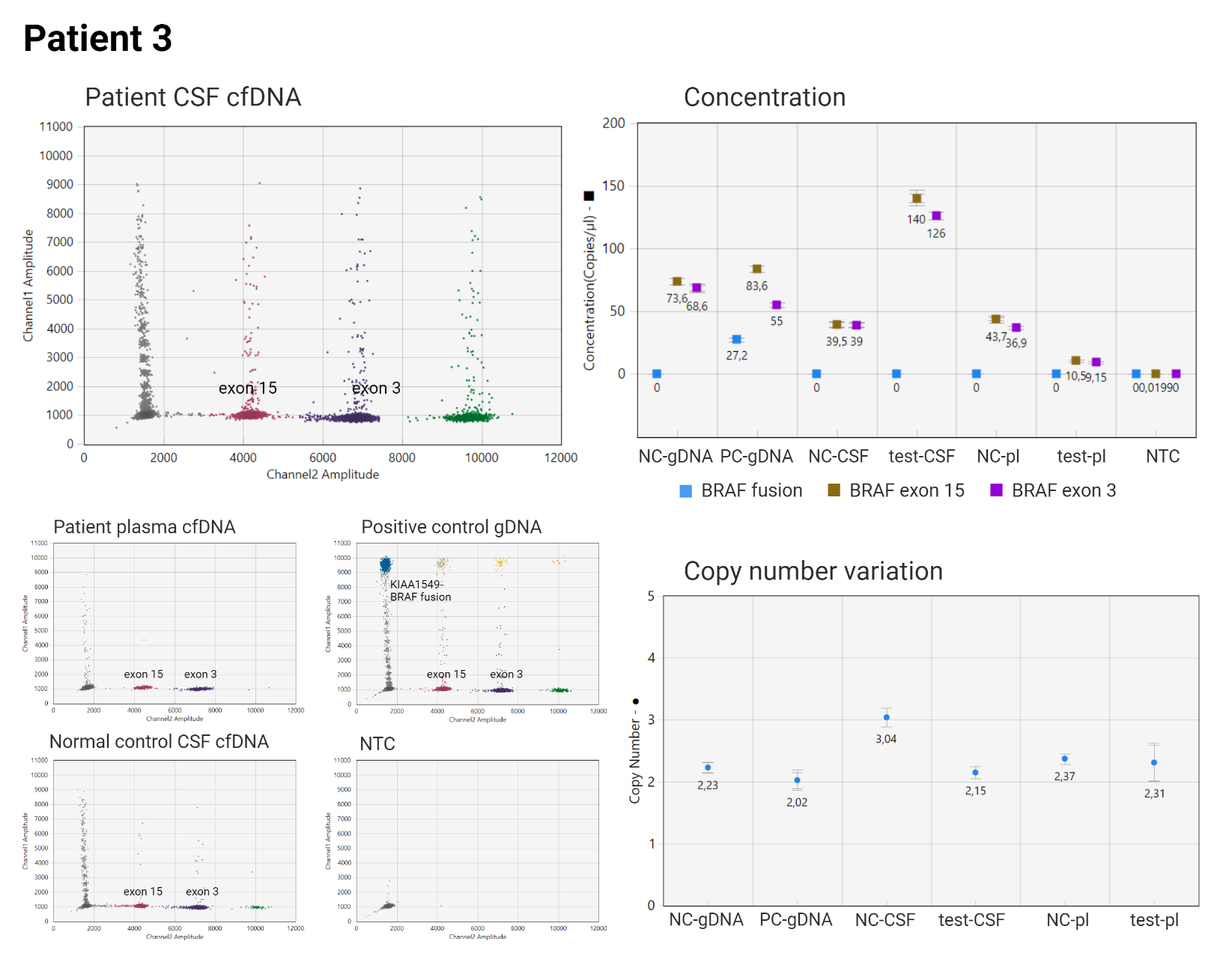


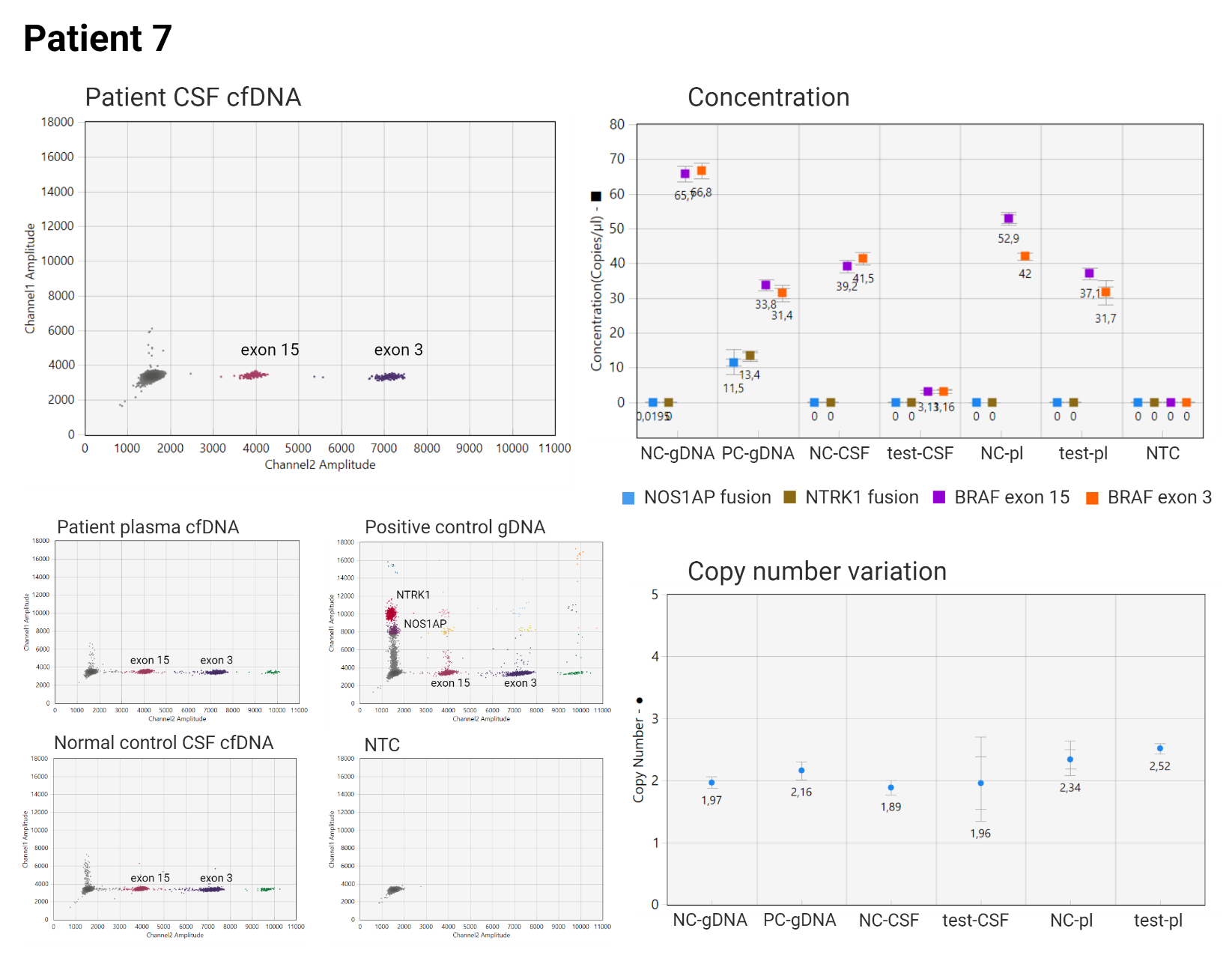


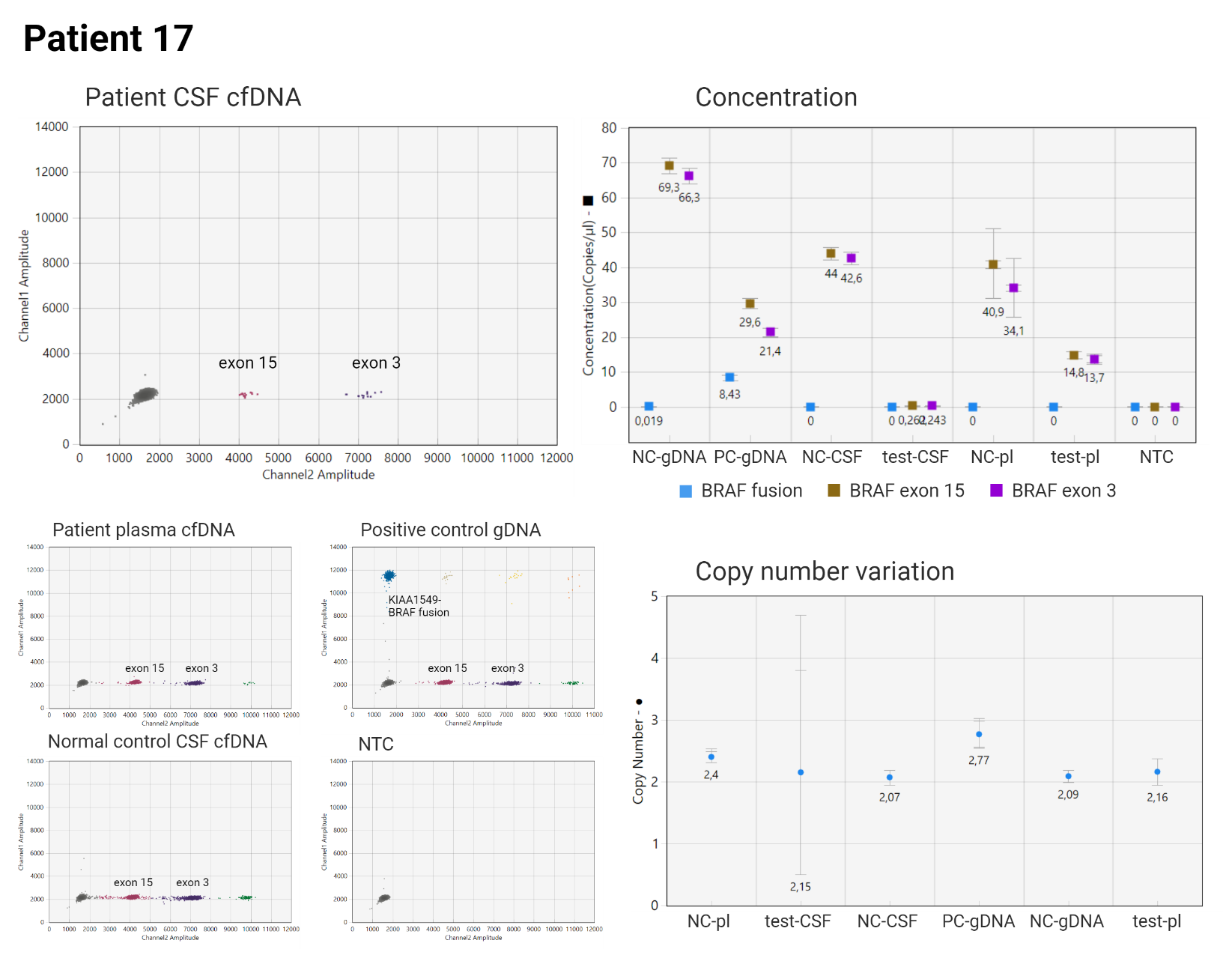


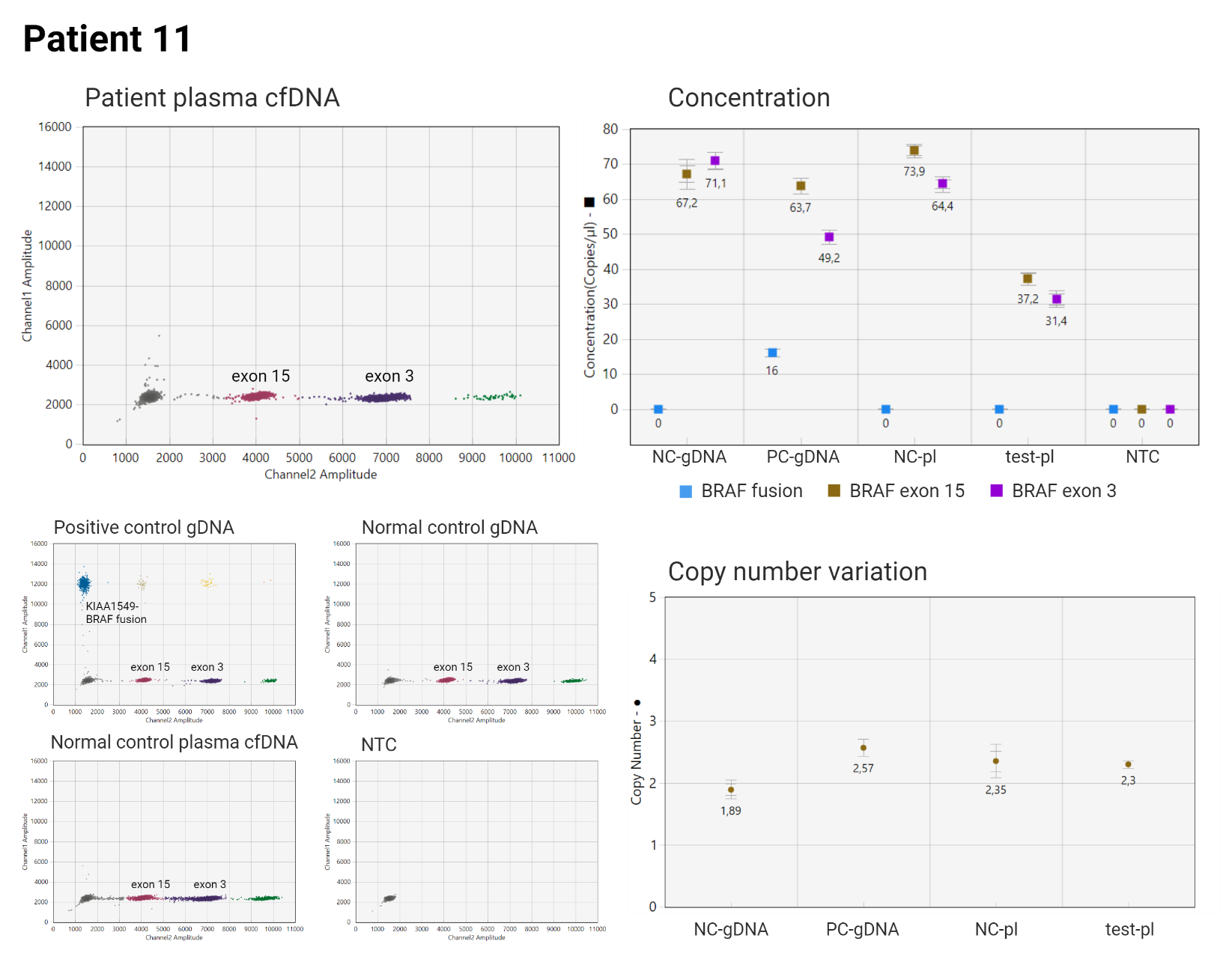


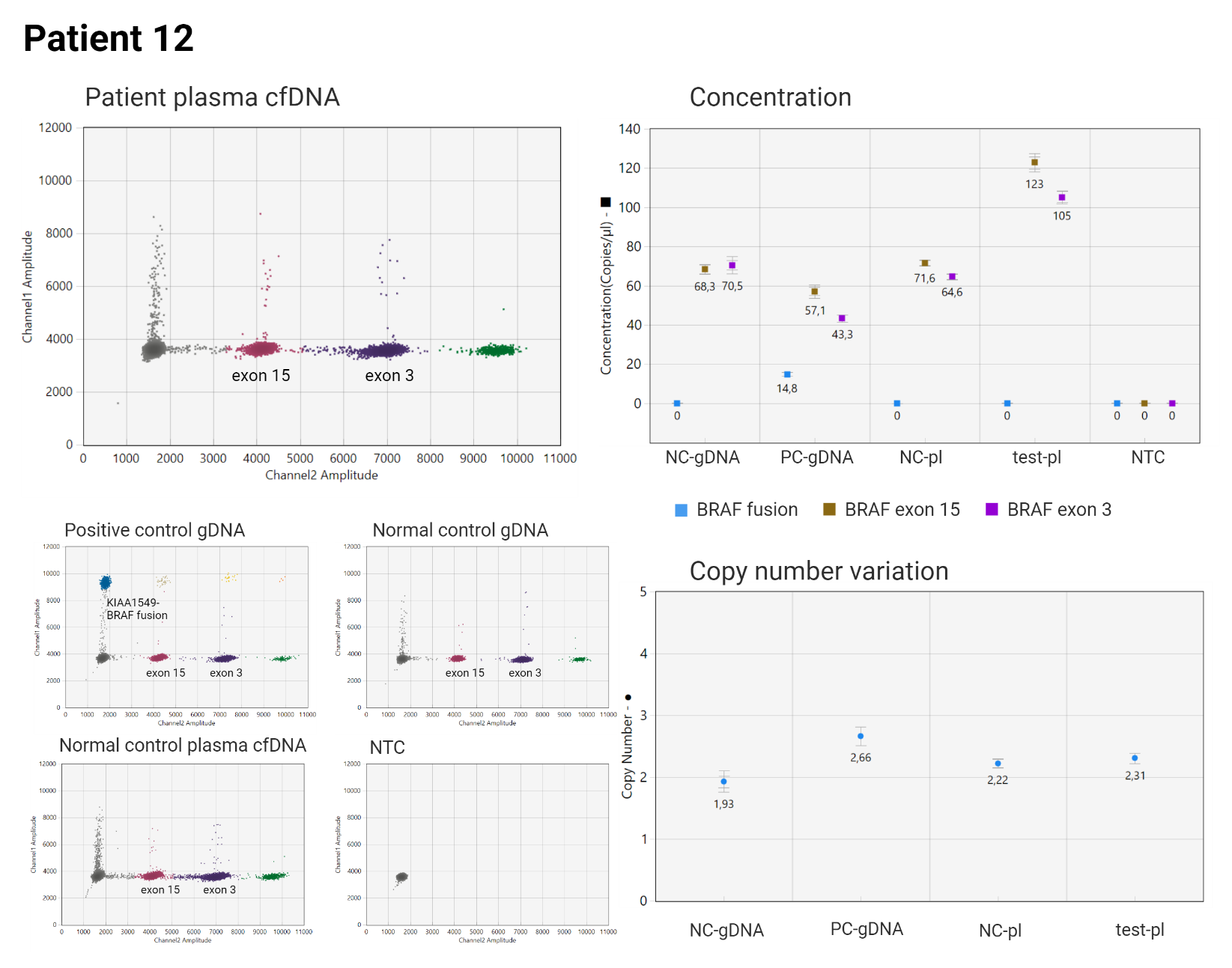


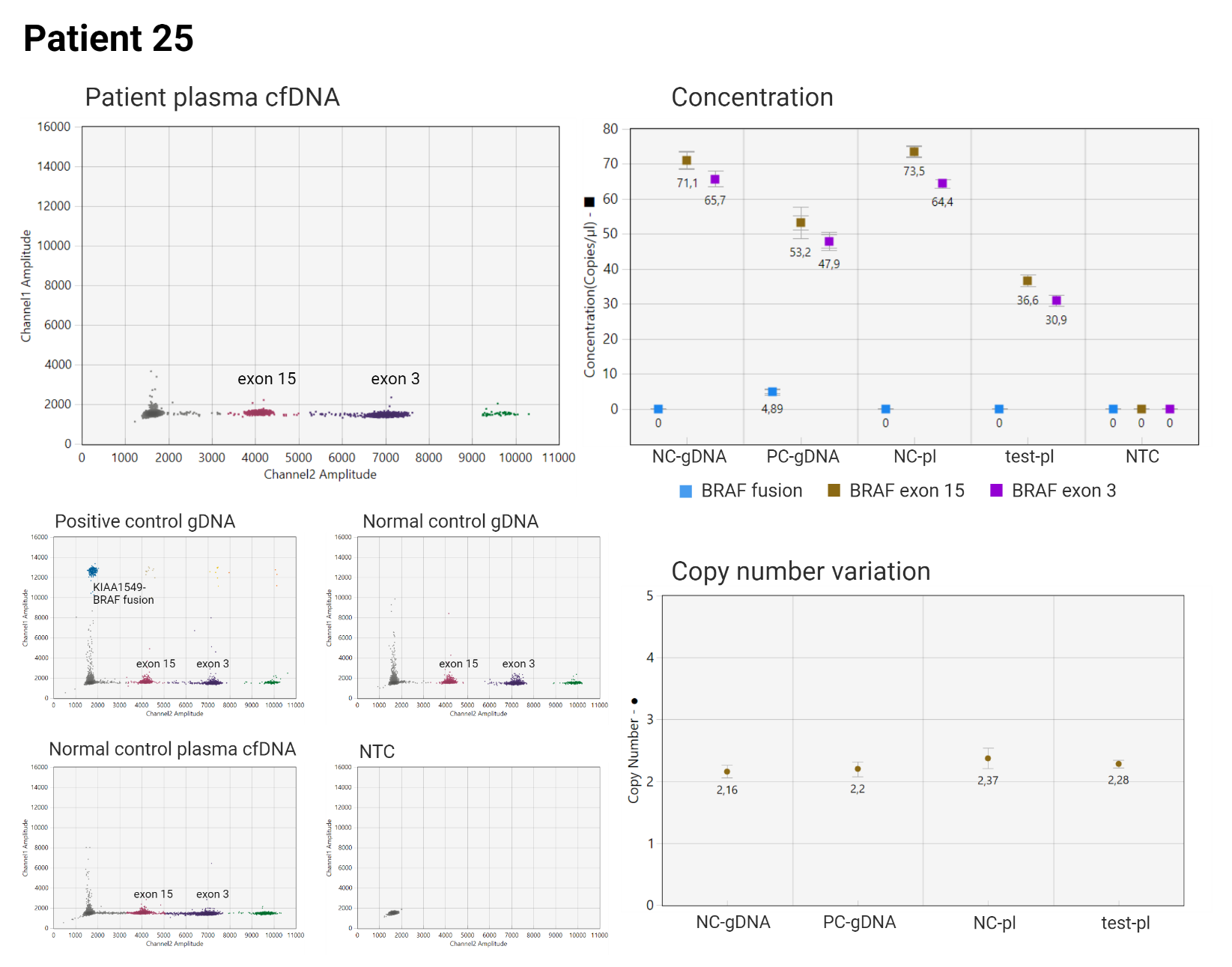

Supplement: vdae008_suppl_Supplementary_Figures_4 [file vdae008_suppl_supplementary_figures_4.docx]
